# OCIAD2 Stabilizes Integrin β1 Signaling Through SNX17‐Mediated Endosomal Recycling to Lipid Rafts and Modulates Cisplatin Response in HNSCC

**DOI:** 10.1002/advs.202515452

**Published:** 2026-02-08

**Authors:** Li Cui, Shanshan Si, Min Ye, Pei Lin, Meiyan Zou, Yunfan Lin, Xu Chen, Bing Guo, Wenjuan Sun, Xinyuan Zhao

**Affiliations:** ^1^ Stomatological Hospital School of Stomatology Southern Medical University Guangzhou Guangdong China; ^2^ School of Dentistry University of California Los Angeles California USA; ^3^ Department of Dentistry The First Affiliated Hospital Sun Yat‐sen University Guangzhou China; ^4^ Department of Stomatology The Third Affiliated Hospital Sun Yat‐sen University Guangzhou China

**Keywords:** cisplatin response, HNSCC, integrin β1, OCIA domain containing 2, SNX17

## Abstract

While cisplatin is a widely used and effective chemotherapeutic agent in the treatment of head and neck squamous cell carcinoma (HNSCC), the molecular mechanisms underlying its resistance remain poorly understood. In this study, we identify OCIA domain containing 2 (OCIAD2) as a central mediator of chemoresistance and tumor progression in HNSCC. Through transcriptomic analysis and Co‐immunoprecipitation coupled with mass spectrometry, we demonstrate that OCIAD2 modulates integrin signaling by directly interacting with integrin β1. Mechanistic investigations reveal that OCIAD2 does not regulate integrin β1 at the transcriptional level, but instead stabilizes its protein expression by preventing lysosomal degradation and enhancing its recycling. Importantly, OCIAD2 binds to SNX17 and enhances its association with integrin β1, promoting its recycling to lipid raft‐enriched regions of the plasma membrane. By maintaining integrin β1 in these lipid raft compartments, OCIAD2 sustains the activation of the FAK–PI3K–AKT–mTOR signaling cascade, thereby fostering cellular resilience and resistance to cisplatin. Moreover, targeting OCIAD2, either through genetic silencing or RNA‐based therapies, significantly sensitizes tumors to cisplatin treatment in preclinical models. This study uncovers a previously unrecognized trafficking‐dependent mechanism of drug resistance, suggesting that OCIAD2 may serve as a novel therapeutic target to overcome chemoresistance in HNSCC.

## Graphical abstract

This study identifies OCIAD2 as a critical regulator of cisplatin resistance in HNSCC. Mechanistically, OCIAD2 stabilizes integrin β1 through a direct physical interaction and facilitates its SNX17‐dependent endosomal recycling to lipid raft microdomains. Targeting OCIAD2 disrupts integrin β1 trafficking and significantly enhances cisplatin sensitivity, supporting OCIAD2 as a potential therapeutic vulnerability for overcoming chemoresistance in HNSCC.

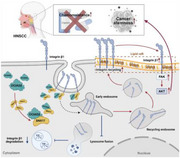

**Abbreviations**
AKT: Akt serine/threonine kinase
ANTs: adjacent normal tissues
CHX: cycloheximide
Co‐IP: co‐immunoprecipitation
CSC: cancer stem cell
DMEM: Dulbecco's modified Eagle medium
FAK: focal adhesion kinase
FDR: false discovery rate
GDC: Genomic Data Commons
GEO: Gene Expression Omnibus
GSEA: gene set enrichment analysis
HNSCC: head and neck squamous cell carcinoma
ITGB1: integrin subunit beta 1
LC‐MS/MS: liquid chromatography‐tandem mass spectrometry
LNP: lipid nanoparticle
mTOR: mechanistic target of rapamycin
NES: normalized enrichment score
NZ: nocodazole
OCIAD2: OCIA domain containing 2
OE: overexpression
PCNA: proliferating cell nuclear antigen
PDX: patient‐derived xenograft
PI3K: phosphatidylinositol 3‐kinase
SNX17: sorting nexin 17
SOX2: SRY‐box transcription factor 2
SRT: short tandem repeat
TCGA: The Cancer Genome Atlas

## 1 Introduction

HNSCC is the sixth most prevalent malignancy globally and is associated with substantial morbidity and mortality [1, 2]. Arising from the mucosal epithelium of the oral cavity, pharynx, larynx, and sinonasal tract, HNSCC encompasses a clinically and molecularly heterogeneous group of tumors with diverse etiologies and outcomes [3, 4, 5]. Despite advances in surgical resection, radiotherapy, and immunotherapy, cisplatin‐based chemotherapy remains a cornerstone of treatment for locally advanced and recurrent or metastatic HNSCC, owing to its DNA crosslinking activity and ability to induce apoptosis in rapidly proliferating tumor cells [6, 7, 8]. However, both intrinsic and acquired resistance to cisplatin severely compromise therapeutic efficacy and are major contributors to disease relapse and poor prognosis [9, 10]. Defining the molecular underpinnings of cisplatin resistance and identifying tractable vulnerabilities represent critical steps toward the development of more durable and effective treatment strategies for HNSCC.

OCIA domain containing 2 (OCIAD2) is a mitochondrial‐associated protein initially identified in ovarian cancer and subsequently implicated in diverse physiological processes, including mitochondrial integrity, activation signaling, and differentiation [11, 12, 13]. Emerging evidences suggest that OCIAD2 also participates in modulating cellular stress responses and signal transduction pathways linked to proliferation and survival [14, 15]. Aberrant expression of OCIAD2 has been reported not only in ovarian neoplasms but also in other malignancies such as lung adenocarcinoma, pancreatic cancer, and glioblastoma, where it contributes to oncogenic signaling and therapeutic resistance [14, 15, 16]. Beyond oncology, OCIAD2 dysregulation has been observed in neurodegenerative diseases, highlighting its broader relevance in pathophysiology [17].

Our transcriptomic analysis revealed a pronounced upregulation of OCIAD2 in cisplatin‐resistant HNSCC patients compared to their sensitive counterparts, highlighting its potential relevance in chemoresistance. Despite these observations, the role of OCIAD2 in HNSCC remains poorly characterized. Deciphering the molecular contributions of OCIAD2 in this context may uncover unrecognized regulatory mechanisms that sustain tumor cell survival under chemotherapeutic stress. Such mechanistic insights could guide the development of targeted strategies to mitigate resistance and improve therapeutic efficacy in HNSCC.

Through integrative transcriptomic profiling, mechanistic dissection, and clinicopathological validation, we demonstrate that OCIAD2 is markedly upregulated in chemoresistant tumors and correlates with aggressive biological behavior and poor clinical outcomes. Mechanistically, OCIAD2 impairs lysosomal degradation of integrin β1 by enhancing the interaction between SNX17 and integrin β1. Specifically, OCIAD2 binds to SNX17 and promotes its association with integrin β1, facilitating its recycling to lipid raft–enriched regions of the plasma membrane. This stabilizes membrane‐localized integrin β1 and sustains activation of the FAK–PI3K–AKT–mTOR signaling cascade, thereby promoting cellular resilience and resistance to cisplatin.

## 2 Materials and Methods

### 2.1 Patient Enrollment and Clinical Specimen Collection

This study adhered to the principles of the Declaration of Helsinki and received approval from the Ethics Committee of the First Affiliated Hospital of Sun Yat‐sen University (IRB Number: 2022‐056). Written informed consent was obtained from all participants. Eligible patients met the following criteria: (1) a histopathological diagnosis of HNSCC and (2) primary surgical treatment. Patients with multiple primary malignancies or incomplete clinical or follow‐up data were excluded.

All patients in this cohort received the same neoadjuvant chemotherapy regimen, consisting of intravenous docetaxel (75 mg/m^2^) and cisplatin (75 mg/m^2^) on day 1, followed by continuous infusion of 5‐fluorouracil (750 mg/m^2^) for 120 h (days 1–5). This cycle was administered twice at 3‐week intervals. Tumor response to neoadjuvant chemotherapy was evaluated through clinical assessment and imaging according to RECIST 1.1 criteria. A complete response (CR) was defined as the disappearance of all target lesions without evidence of disease elsewhere. A partial response (PR) required at least a 30% reduction in the sum of the longest diameters of target lesions. Progressive disease (PD) was defined as a ≥ 20% increase in the sum of the longest diameters (with an absolute increase of ≥ 5 mm) compared with the smallest value recorded since treatment initiation, or the appearance of new lesions. Stable disease (SD) was assigned when the extent of change did not meet criteria for PR or PD. Patients achieving CR or PR were classified as chemotherapy‐sensitive, whereas those exhibiting SD or PD were categorized as chemotherapy‐resistant. Tissue specimens were either snap‐frozen in liquid nitrogen and stored at −80°C or fixed in formalin and embedded in paraffin. A total of 135 patients were enrolled in this in‐house HNSCC cohort. All enrolled patients met the predefined inclusion criteria and were included in subsequent analyses, with no exclusions after enrollment. The clinicopathological characteristics of the in‐house HNSCC cohort are summarized in Table S1.

### 2.2 Cell Lines and Cell Culture

The UMSCC‐1 (SCC‐1; HPV‐negative) and UMSCC‐23 (SCC‐23; HPV31‐positive) cell lines were obtained from the University of Michigan, whereas the human foreskin fibroblast cell line HFF‐1 was sourced from the American Type Culture Collection (ATCC). Cells were cultured in Dulbecco's Modified Eagle Medium supplemented with 10% fetal bovine serum, 100 U/mL penicillin, and 100 µg/mL streptomycin, and maintained at 37°C in a humidified incubator with 5% CO_2_. Cell line authentication was confirmed by STR profiling. All cell lines were regularly tested for mycoplasma contamination and confirmed to be negative. For in vitro drug treatment experiments, cisplatin was used at a final concentration of 5 µm. Cells were treated immediately after medium replacement and continuously exposed to cisplatin until the indicated experimental endpoints, unless otherwise specified.

### 2.3 Plasmid, Lentivirus, and the Transfection

To establish stable gene knockdown, shRNA oligonucleotides (GenePharma, Shanghai, China) targeting *OCIAD2*, *SNX17*, or *ITGB1* were cloned into the LV3‐pGLV‐h1‐GFP‐puro lentiviral vector (GenePharma). For overexpression, full‐length cDNAs of *OCIAD2* and *SNX17* were inserted into the pGCL‐GFP lentiviral backbone (Genechem, Shanghai, China). Lentiviral particles were produced by co‐transfecting HEK293T cells with the expression plasmids (Genechem) and packaging plasmids (Genechem). After 72 h, viral supernatants were collected, filtered through 0.45 µm membranes, and concentrated by ultracentrifugation. HNSCC cells were transduced with the indicated lentiviruses at appropriate multiplicities of infection, followed by puromycin selection to establish stable cell lines. The target sequences of shRNAs were listed in Table S2. C‐terminally Flag‐tagged full‐length OCIAD2 and its truncations (aa 1–50, 51–100, 101–154) were cloned into KpnI/EcoRI‐digested pcDNA3.1(+) (Invitrogen, Carlsbad, CA, USA). N‐terminally HA‐tagged full‐length integrin β1 and its deletion mutants (aa 1–100 to 701–798) were cloned into BamHI/EcoRI‐digested pcDNA3.1(+) (Invitrogen). The myristoylated AKT (Myr‐AKT) construct was obtained from Addgene (Cambridge, MA, USA). Plasmids were transfected using Lipofectamine 3000 (Invitrogen) according to the manufacturer's protocol.

### 2.4 Quantitative Real‐Time PCR

Total RNA was extracted from cells using the Quick‐RNA kit (Zymo Research Corp, Irvine, CA, USA), and cDNA was synthesized using SuperScript III First‐Strand Synthesis SuperMix (Invitrogen, Carlsbad, CA, USA) according to the manufacturer's instructions. Quantitative real‐time PCR was performed on a CFX96 Real‐Time PCR detection system (Bio‐Rad, Hercules, CA, USA) using LightCycler 480 SYBR Green I MasterMix (Roche Applied Science, Indianapolis, IN, USA). Gene expression levels were quantified by the 2^−ΔΔCt^ method, with β‐actin serving as an internal control for normalization. The primers used for qRT‐PCR are listed in Table S2.

### 2.5 Western Blotting

Equal amounts of protein samples were resolved on 4%–20% SDS‐PAGE gels and transferred to PVDF membranes using a Trans‐Blot Turbo system (Bio‐Rad). Membranes were blocked with protein‐free rapid blocking buffer (Epizyme, Shanghai, China) for 10 min at room temperature, followed by incubation with primary antibodies at 4°C overnight. After washing the membranes five times with TBST for 5 min each, membranes were incubated with HRP‐conjugated secondary antibodies (Proteintech, Chicago, IL, USA) for 1 h at room temperature. Following additional washes, protein bands were visualized using Amersham ECL Prime Western Blotting Detection Reagent (Cytiva, Marlborough, MA, USA). The primary antibodies used in this study are as follows: OCIAD2 (Abcam, Cambridge, UK), CD133 (Proteintech), CD44 (Proteintech), SOX2 (Proteintech), BMI‐1 (Proteintech), p‐FAK (Cell Signaling Technology, Danvers, MA, USA), FAK (Proteintech), p‐PI3K (Thermo Fisher Scientific,), PI3K (Thermo Fisher Scientific), p‐AKT (Proteintech), AKT (Proteintech), p‐mTOR (Proteintech), mTOR (Proteintech), PCNA (Proteintech), E‐cadherin (Proteintech), N‐cadherin (Proteintech), integrin β1 (Proteintech), FLOT2 (Thermo Fisher Scientific), SNX17 (Abcam), EpCAM (Proteintech), α‐SMA (Proteintech), cytokeratin 14 (ABclonal, Wuhan, China) and β‐actin (Proteintech). Anti‐HA and anti‐FLAG tag antibodies were obtained from Proteintech.

### 2.6 Immunohistochemistry Analysis

Paraffin‐embedded tissue sections were first deparaffinized and rehydrated through a graded ethanol series. To prevent nonspecific binding, sections were blocked with goat serum for 2 h at room temperature, followed by overnight incubation at 4°C with the primary antibodies. After rinsing with PBS, sections were exposed to HRP‐conjugated secondary antibodies for 1 h at room temperature. Immunoreactivity was detected using a DAB chromogenic substrate. For semi‐quantitative analysis, immunostaining was evaluated using the H‐score method, which integrates both staining intensity and the proportion of positive cells. Staining was graded on a 4‐point scale: 0 (no staining), 1 (low intensity), 2 (intermediate), and 3 (high intensity). The H‐score was calculated as follows: (1 × percentage of low‐intensity cells) + (2 × percentage of intermediate‐intensity cells) + (3 × percentage of high‐intensity cells), resulting in a final score ranging from 0 to 300.

### 2.7 Immunofluorescence and Colocalization Analysis

Cells were fixed with 4% paraformaldehyde for 10 min and permeabilized with 0.1% Triton X‐100 for 10 min at room temperature, followed by three PBS washes. After blocking with immunostaining blocking buffer (Beyotime) for 1 h, cells were incubated overnight at 4°C with primary antibodies against OCIAD2 and integrin β1. Following incubation with appropriate fluorophore‐conjugated secondary antibodies for 1 h at room temperature, nuclei were counterstained with DAPI for 5 min. Fluorescence images were acquired using a Leica STELLARIS 5 confocal microscope (Leica Microsystems, Wetzlar, Germany), and colocalization analysis was performed based on fluorescence signal overlap.

### 2.8 Colony Formation Assay

Cells were seeded at a density of 3000 cells per well in six‐well plates and cultured for two weeks. Colonies were fixed with 4% paraformaldehyde and stained with 0.5% crystal violet. Plates were then imaged, and the relative colony area was quantified using ImageJ software.

### 2.9 Matrigel‐Coated Transwell Invasion Assay

Cell invasion was assessed using Matrigel‐coated Transwell inserts (8 µm pore size; Costar, Cambridge, MA, USA) placed in 24‐well plates. Following overnight serum starvation, cells were resuspended in serum‐free medium and seeded into the upper chambers at a density of 5 × 10^5^ cells per insert. The lower chambers were filled with complete medium as a chemoattractant. After an appropriate incubation period allowing for cell invasion, non‐migrated cells on the upper membrane surface were removed with a cotton swab. Invaded cells on the underside were fixed with 4% paraformaldehyde and stained with 0.5% crystal violet. Images from six random fields per insert were acquired, and the average invaded area per field was quantified. The average invaded area per field was quantified using ImageJ. First, the images were imported into ImageJ and converted to an 8‐bit grayscale mode. Then, the “Adjust” menu was used to apply the “Threshold” function, which allowed for the calculation of the invaded area. The area was measured by selecting the “Analyze” menu and then using the “Measure” function to quantify the invaded region.

### 2.10 Tumor Sphere Formation Assay

Single‐cell suspensions were seeded into ultralow‐attachment 96‐well plates (Corning) at 500 cells per well and cultured in serum‐free DMEM/F12 medium (Gibco) supplemented with 1% B27 (Invitrogen), 1% N2 (Invitrogen), 20 ng/mL EGF (PeproTech), and 10 ng/mL bFGF (PeproTech). Cultures were maintained under these conditions until spheroid structures became evident. Tumor sphere‐forming capacity was quantified by measuring the sphere formation area as a percentage of the total well area using ImageJ.

### 2.11 Annexin V/PI Apoptosis Assay

Cells subjected to the indicated treatments were detached using trypsin, washed with PBS, and resuspended in binding buffer. Annexin V conjugated to APC (Thermo Fisher Scientific) was added, and samples were incubated for 15 min at room temperature in the dark. After staining, cells were centrifuged, resuspended in fresh binding buffer, and propidium iodide was added immediately before flow cytometric analysis. Apoptotic cells were analyzed within 30 min using a DxFLEX flow cytometer (Beckman Coulter, Inc., Brea, CA, USA).

### 2.12 RNA Sequencing

RNA sequencing was conducted on tumor samples obtained from eight cisplatin‐resistant (*n* = 8) and eight cisplatin‐sensitive (*n* = 8) patients, as well as on SCC‐1 cells transduced with lentiviruses expressing either shCTRL or shOCIAD2. Total RNA was extracted using TRIzol reagent (Invitrogen, Waltham, USA), and sequencing was performed on the BGISEQ‐500 platform at the Beijing Genomics Institute (Beijing, China). Sequencing reads were aligned to the human reference genome (GRCh37/hg19) using the HISAT pipeline (http://www.ccb.jhu.edu/software/hisat/). Clean reads were mapped to annotated genes with Bowtie2, and gene expression levels were quantified using the RNA‐Seq by Expectation‐Maximization (RSEM) algorithm. Differentially expressed genes were identified using DEGseq, with thresholds set at fold change ≥ 2 or ≤ 0.5 and adjusted *p* value < 0.05. Gene Set Enrichment Analysis (GSEA) was performed to identify enriched pathways between shOCIAD2 and shCTRL groups based on RNA‐seq expression profiles. Normalized enrichment scores (NES) and false discovery rates (FDR) were calculated to assess statistical significance.

### 2.13 Co‐Immunoprecipitation (Co‐IP) Assay

Cells were lysed in RIPA buffer on ice for 30 min. After centrifugation at 14 000 × g for 20 min at 4°C, the supernatants were collected and incubated with the appropriate antibodies overnight at 4°C with gentle rotation. The immune complexes were captured using magnetic beads and incubated for an additional 6–8 h at 4°C. Beads were washed thoroughly with buffer solution, and the bound proteins were eluted by boiling in SDS sample buffer at 100°C for 10 min. The immunoprecipitates were analyzed by immunoblotting.

### 2.14 Liquid Chromatography–Tandem Mass Spectrometry (LC‐MS/MS)

Proteins interacting with OCIAD2 were separated by SDS‐PAGE, and selected gel bands were excised and subjected to in‐gel trypsin digestion. The resulting peptides were analyzed using a Thermo Scientific Easy‐nLC 1200 system coupled to an Orbitrap Exploris 480 mass spectrometer (Thermo Fisher Scientific) operated in data‐dependent acquisition mode. Protein identification was performed by searching the acquired spectra against a reference database.

### 2.15 Cycloheximide (CHX) Chase Assay

To assess the impact of OCIAD2 on the stability of integrin β1, cells with either OCIAD2 overexpression or depletion were treated with CHX at a final concentration of 100 µg/mL to inhibit de novo protein synthesis. At the indicated time points following CHX treatment, cells were harvested, and total protein lysates were extracted. Integrin β1 protein levels were examined by western blotting to determine their degradation kinetics under different OCIAD2 expression conditions.

### 2.16 Sucrose Density Gradient Fractionation

Lipid raft fractions were isolated using sucrose density gradient centrifugation. Briefly, cells were lysed under the indicated treatment conditions, and the lysates were carefully layered onto a discontinuous sucrose gradient prepared with varying sucrose concentrations. Samples were centrifuged at 44 000 rpm for 18 h at 4°C. After centrifugation, twelve sequential fractions were collected from the top to the bottom of the gradient and analyzed by western blotting.

### 2.17 Flow Cytometric Analysis of Integrin β1 Surface Expression

To assess integrin β1 trafficking, HNSCC cells were serum‐starved for 12 h and treated with nocodazole to depolymerize microtubules. The drug was then removed by washing with serum‐free medium, allowing microtubule repolymerization to proceed for the indicated time intervals. At each time point following nocodazole washout, cells were collected and incubated with PE‐conjugated anti‐β1 integrin antibody (1:150; eBioscience, San Diego, CA, USA) at 4°C for 30 min. After removal of unbound antibodies by washing, cells were fixed and analyzed using a DxFLEX flow cytometer (Beckman Coulter, Inc.) to quantify surface integrin β1 expression based on mean fluorescence intensity.

### 2.18 Integrin β1 Internalization and Recycling Assay

To evaluate the trafficking behavior of integrin β1, surface proteins were labeled with cleavable biotin using the Cell Surface Protein Biotinylation and Isolation Kit (Thermo Fisher Scientific) according to the manufacturer's instructions. After internalization at 37°C for defined intervals, remaining surface biotin was selectively removed with a membrane‐impermeable stripping buffer. For recycling analysis, cells were incubated with or without stripping treatment after internalization. Biotinylated proteins were enriched using streptavidin‐conjugated beads and analyzed by immunoblotting to determine the levels of internalized and recycled integrin β1.

### 2.19 In Vivo Subcutaneous Tumor Model

All animal experiments were conducted in accordance with the guidelines approved by the IACUC of Southern Medical University (SMUL202403025). Six‐week‐old male BALB/c nude mice with a body weight of 18–21 g were used to establish subcutaneous tumor models. A 100 µL suspension containing 2 × 10^7^ cells/mL was injected into the dorsal flank of each mouse. For in vivo studies, cisplatin was administered intraperitoneally at a dose of 3 mg/kg once weekly for three consecutive weeks in the designated treatment groups. At the experimental endpoint, mice were euthanized, and tumors were excised for measurement of tumor weight and volume. Tumor tissues were then fixed in formalin, paraffin‐embedded, and processed for IHC analysis.

### 2.20 PDX‐Based Analysis of Cancer Stemness and Response to OCIAD2 Inhibition

Patient‐derived xenograft (PDX) mouse models were established as previously described. Briefly, small fragments (2–3 mm^3^) of surgical specimens from primary HNSCC tumors were implanted subcutaneously into male NSG mice within 4 h of resection. Tumor growth, body weight, and overall health were regularly monitored. Once tumors reached approximately 1 cm^3^, they were excised, and the mice were euthanized. Tumor tissues were serially transplanted into new NSG mice to generate passage 1 (P1) PDX tumors, and this process was repeated to establish passage 2 (P2) tumors. Distinct PDX models, each derived from a different HNSCC patient, were created for further analysis.

Primary tumor cells from cisplatin‐resistant and cisplatin‐sensitive PDX tumors were isolated and implanted into mice for in vivo limiting dilution assays. Briefly, freshly excised PDX tumor tissues were minced into small fragments and enzymatically dissociated using collagenase and DNase I. Single‐cell suspensions were obtained by filtration through a 70‐µm cell strainer. To confirm the epithelial origin of the isolated cells and minimize stromal contamination, cell lysates were analyzed by immunoblotting for epithelial markers (CK14 and EpCAM) and the mesenchymal marker α‐SMA. Only epithelial marker–positive, α‐SMA–negative cell populations were used for subsequent in vivo experiments. The CSC frequency was determined using the Extreme Limiting Dilution Analysis software (https://bioinf.wehi.edu.au/‐software/elda/).

To assess the therapeutic relevance of targeting OCIAD2, male PDX‐bearing mice were randomized when tumor volumes reached approximately 50 mm^3^. Mice were treated with lipid nanoparticle–formulated siRNA targeting OCIAD2 (LNP‐siOCIAD2; RiboBio, Guangzhou, China), cisplatin, or a combination of both. LNP‐siRNAs were administered intravenously, and cisplatin was delivered intraperitoneally. Tumor growth was monitored every three days, and tumors were harvested at the endpoint for further analysis. Systemic safety was evaluated by monitoring body weight, measuring serum biochemical markers of hepatic and renal function, and performing histological examination of major organs.

### 2.21 Analysis of the Clinical Relevance of *OCIAD2* Using Public Databases

To explore the clinical relevance of OCIAD2 in HNSCC, several publicly available datasets were analyzed. Gene expression profiles comparing HNSCC tissues with normal or precancerous tissues were obtained from the NCBI Gene Expression Omnibus (GEO) database, including GSE127165, GSE58911, GSE37991, GSE143224, GSE25099, GSE31056, and GSE30784. Additionally, GSE41613, a microarray dataset with corresponding clinical annotations, was retrieved for survival analysis. RNA sequencing data and matched clinical information for all tumor types were downloaded from The Cancer Genome Atlas (TCGA) via the Genomic Data Commons (GDC) portal (https://gdc.cancer.gov/). To perform survival analysis, patients were stratified based on *OCIAD2* expression levels. The optimal cutoff for dividing patients into high and low *OCIAD2* expression groups was determined using X‐tile software (https://medicine.yale.edu/lab/rimm/research‐/software /).

### 2.22 Statistical Analysis

All statistical analyses were performed using GraphPad Prism 9.0 (GraphPad Software, San Diego, CA, USA). Data distribution normality was assessed using the Shapiro–Wilk test, and parametric or nonparametric statistical methods were applied accordingly. Data were expressed as the mean ± standard deviation. Statistical significance was assessed using one‐way ANOVA or Student's *t*‐test, as appropriate. Spearman correlation analysis was conducted to evaluate the relationship between OCIAD2 and integrin β1 expression. Survival analysis was performed using the Kaplan–Meier method, with differences assessed by the log‐rank test. Multivariate analysis was used to identify independent prognostic factors associated with overall survival. A *p*‐value of < 0.05 was considered statistically significant.

## 3 Results

### 3.1 OCIAD2 Confers Chemoresistance and Is Associated with Adverse Clinical Outcomes in HNSCC

**FIGURE 1:**
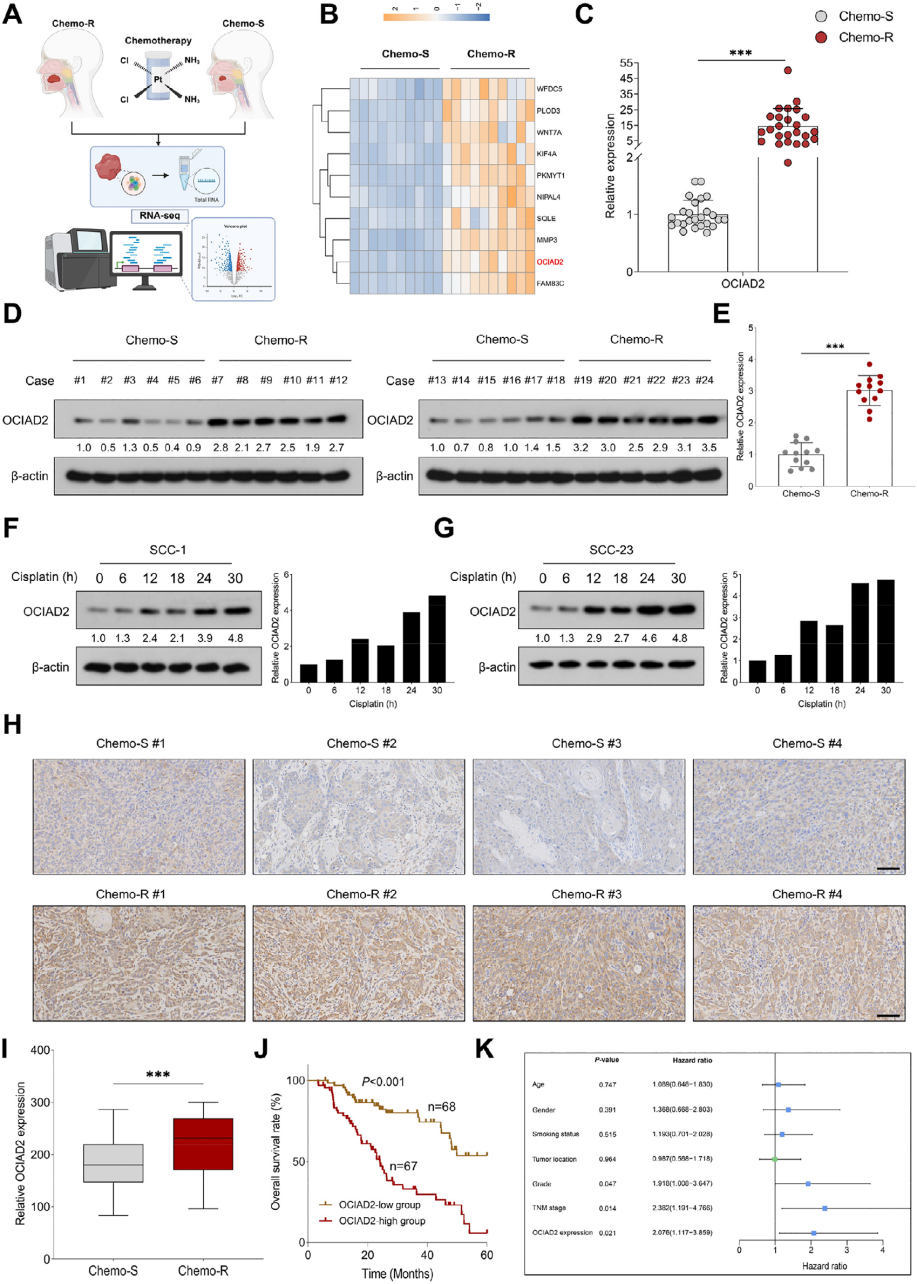
OCIAD2 as a crucial modulator of chemoresistance in HNSCC. (A) Schematic representation of the transcriptome sequencing workflow for chemotherapy‐sensitive (Chemo‐S) and chemotherapy‐resistant (Chemo‐R) patient samples. (B) Heatmap showing the most differentially expressed genes between Chemo‐S (*n* = 10) and Chemo‐R (*n* = 10) patients. (C) Expression levels of *OCIAD2* mRNA in tumor samples from Chemo‐S (*n* = 25) and Chemo‐R (*n* = 25) patients. (D,E) Western blot analysis of OCIAD2 protein expression in Chemo‐S (*n* = 12) and Chemo‐R (*n* = 12) tumors. (F,G) Time‐course analysis of OCIAD2 protein expression in SCC‐1 and SCC‐23 cells following cisplatin treatment at the indicated time points (*n* = 3). (H,I) IHC analysis of OCIAD2 staining intensity in tumor tissues from Chemo‐S and Chemo‐R patients. Scale bar, 100 µm. (J) Kaplan–Meier survival curve for overall survival in HNSCC patients (*n* = 135) stratified by median OCIAD2 expression; survival differences were assessed using the log‐rank test. (K) Multivariate Cox proportional hazards regression analysis identifying independent risk factors for overall survival in HNSCC. Data are presented as mean ± SD unless otherwise indicated. Two‐group comparisons were performed using a two‐tailed unpaired Student's *t*‐test. For comparisons among multiple groups, one‐way ANOVA with post hoc multiple‐comparisons testing was used. OE=overexpression. ^***^ *p* < 0.001.

To identify oncogenic drivers underlying chemoresistance in HNSCC, we performed transcriptomic profiling of tumor specimens from chemotherapy‐sensitive (Chemo‐S) and chemotherapy‐resistant (Chemo‐R) patients. This analysis revealed OCIAD2 as one of the most significantly upregulated genes in the Cis‐R group (Figure 1A,B). Validation by qRT‐PCR and immunoblotting confirmed a marked increase in OCIAD2 expression in Chemo‐R tumors compared to Chemo‐S counterparts (Figure 1C,E). In vitro, exposure of SCC‐1 and SCC‐23 cells to cisplatin led to a time‐dependent accumulation of OCIAD2 protein, suggesting that chemotherapeutic stress may itself contribute to its upregulation (Figure 1F,G). Consistently, immunohistochemical staining of clinical specimens demonstrated significantly stronger OCIAD2 expression in Chemo‐R tumors (Figure 1H,I).

To assess the broader relevance of these findings, we examined multiple independent public datasets. Across six cohorts—GSE127165, GSE58911, GSE37991, TCGA‐HNSCC, GSE143224, and GSE25099—*OCIAD2* expression was consistently elevated in tumor tissues relative to adjacent normal tissues (ANTs) or normal controls (Figure S1A–F). Similar trends were observed in the GSE31056 cohort, where tumor tissues exhibited higher *OCIAD2* levels compared to both normal mucosa and surgical margins (Figure S1G). In the GSE30784 dataset, *OCIAD2* expression increased progressively from normal mucosa to dysplastic lesions and carcinoma, suggesting a potential role in tumor initiation and progression (Figure S1H).

Survival analysis of the TCGA HNSCC and GSE41613 cohorts revealed that patients with high OCIAD2 expression had significantly worse overall survival compared to those with low expression (Figure S1I,J). This prognostic association was further validated in our in‐house HNSCC cohort, where elevated OCIAD2 staining intensities were also significantly correlated with poor overall survival (Figure 1J).

Consistent with these findings, stratified Kaplan–Meier analyses demonstrated that high OCIAD2 expression was associated with significantly reduced overall survival in both smokers and non‐smokers (Figure S1K,L), indicating that the adverse prognostic impact of OCIAD2 is maintained across distinct smoking status subgroups and is not solely attributable to smoking‐related risk. More importantly, multivariate Cox proportional hazards regression analysis, adjusted for conventional prognostic variables including age, gender, smoking status, tumor site, grade, and TNM stage, identified OCIAD2 as an independent predictor of overall survival (Figure 1K).

### 3.2 OCIAD2 Modulates Cisplatin Response by Sustaining Stem‐Like Traits in HNSCC

**FIGURE 2:**
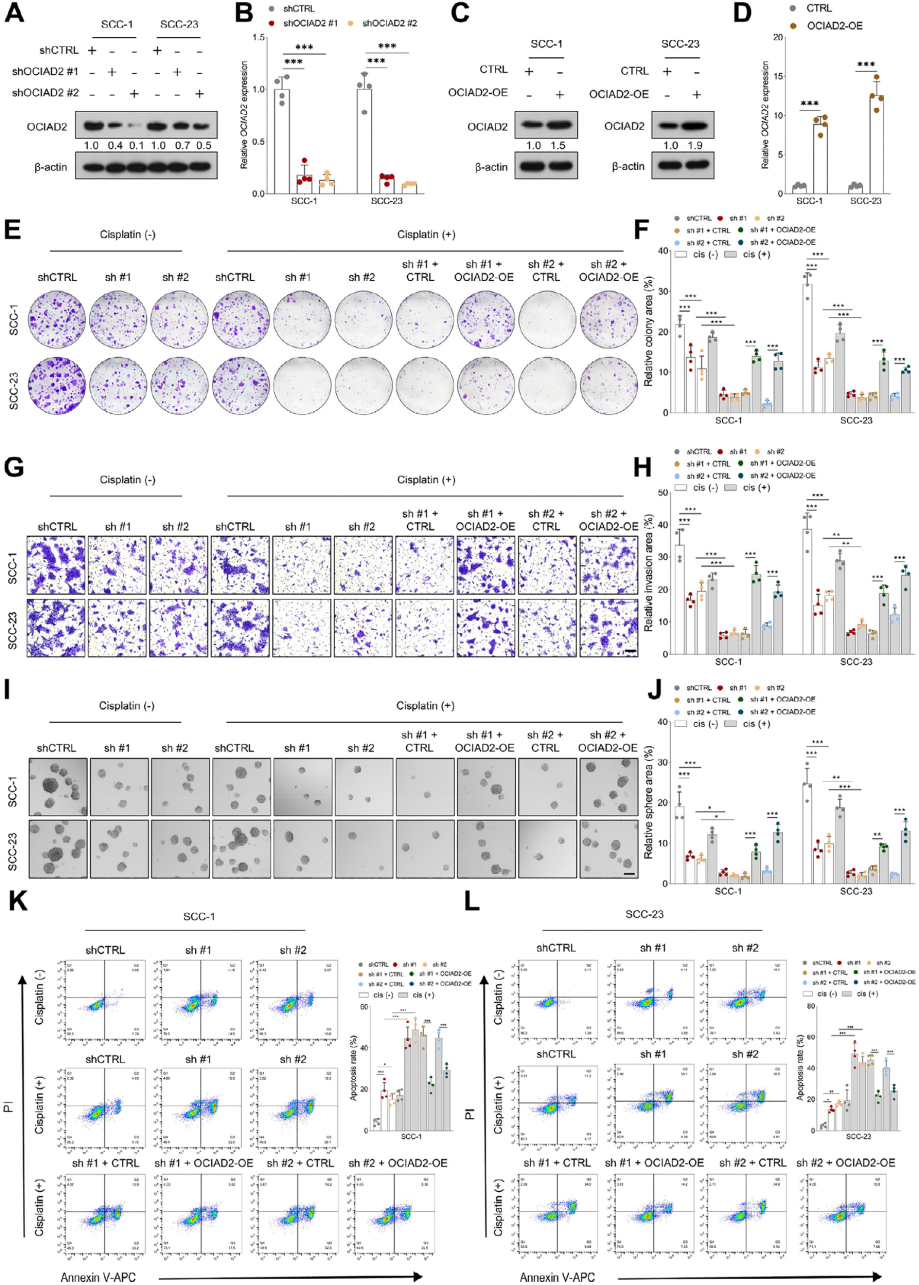
OCIAD2 drives adaptive phenotypic remodeling in response to cisplatin in HNSCC. (A,B) Western blot analysis (*n* = 3) and qRT‐PCR (*n* = 4) quantification of OCIAD2 expression in SCC‐1 and SCC‐23 cells following OCIAD2 knockdown. (C,D) Western blot (*n* = 3) and qRT‐PCR (*n* = 4) analysis of OCIAD2 expression in SCC‐1 and SCC‐23 cells with OCIAD2 overexpression or control treatment. (E,F) Colony formation assays of SCC‐1 and SCC‐23 cells stably transduced with control vector, OCIAD2‐targeting short hairpin RNAs, or combined knockdown and OCIAD2 re‐expression, cultured in the absence or presence of cisplatin (*n* = 4). (G,H) Transwell invasion assays of SCC‐1 and SCC‐23 cells subjected to OCIAD2 knockdown, knockdown with re‐expression, or control treatment, in the presence or absence of cisplatin (*n* = 4). Scale bar, 100 µm. (I,J) Tumorsphere formation assays assessing the self‐renewal capacity of SCC‐1 and SCC‐23 cells following OCIAD2 knockdown or rescue, with or without cisplatin exposure (*n* = 4). Scale bar, 100 µm. (K,L) Flow cytometric analysis of apoptosis in SCC‐1 and SCC‐23 cells after OCIAD2 knockdown or rescue, treated with or without cisplatin, using Annexin V–APC and propidium iodide staining (*n* = 4). Data are presented as mean ± SD unless otherwise indicated. Two‐group comparisons were performed using a two‐tailed unpaired Student's *t*‐test. For comparisons among multiple groups, one‐way ANOVA with post hoc multiple‐comparisons testing was used. OE=overexpression. ^*^ *p* < 0.05, ^**^ *p* < 0.01 and ^***^ *p* < 0.001.

To investigate the functional role of OCIAD2 in cisplatin responsiveness, we manipulated its expression in HNSCC cell lines using lentivirus‐mediated shRNA knockdown and overexpression constructs. Western blotting and qRT‐PCR confirmed efficient silencing and enforced expression of OCIAD2 in both SCC‐1 and SCC‐23 cells (Figure 2A–D). Functionally, OCIAD2 depletion led to reduced clonogenicity even in the absence of cisplatin and further potentiated the inhibitory effect of cisplatin on colony formation. Conversely, ectopic OCIAD2 expression partially rescued the clonogenic capacity of cells exposed to cisplatin, suggesting that OCIAD2 confers a survival advantage under chemotherapeutic stress (Figure 2E–F). These findings were corroborated by transwell invasion and tumorsphere assays, in which OCIAD2 knockdown synergized with cisplatin to suppress both invasive behavior and self‐renewal capacity, while OCIAD2 overexpression attenuated these effects (Figure 2G–J). Flow cytometric analysis further demonstrated that silencing OCIAD2 markedly increased cisplatin‐induced apoptosis, while its overexpression attenuated apoptotic responses (Figure 2K,L).

**FIGURE 3:**
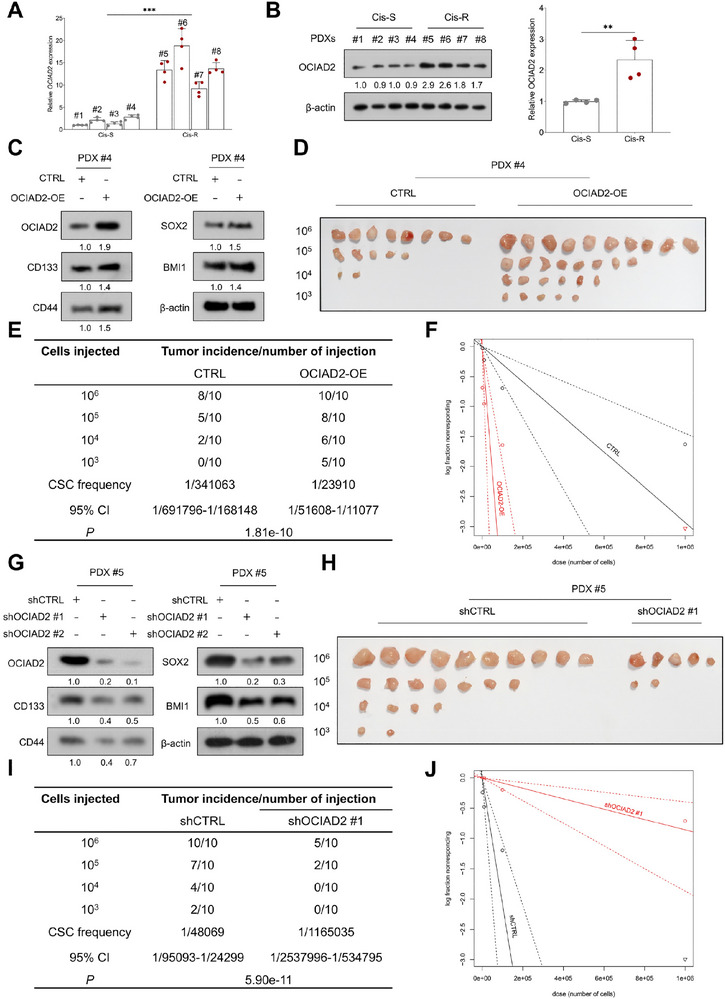
OCIAD2 drives tumor‐initiating capacity in PDX models of HNSCC. (A,B) Quantitative RT‐PCR (*n* = 4) and immunoblot (*n* = 3) analysis of OCIAD2 mRNA and protein expression in Cisplatin‐S and Cisplatin‐R PDX tumor tissues. (C) Immunoblot analysis of CD133, CD44, SOX2, and BMI‐1 expression in primary HNSCC cells derived from PDX#4 following OCIAD2 overexpression (*n* = 3). (D–F) In vivo limiting dilution assays of PDX#4‐derived HNSCC cells transduced with control or OCIAD2‐overexpressing lentiviruses. Cells were injected at four different doses, with *n* = 10 mice per dose. CSC frequency was estimated based on tumor formation rates at each injected cell dose using ELDA. (G) Immunoblot analysis of CD133, CD44, SOX2, and BMI‐1 levels in PDX#5‐derived HNSCC cells following OCIAD2 knockdown (*n* = 3). (H–J) In vivo limiting dilution assays of PDX#5‐derived HNSCC cells transduced with control or OCIAD2‐silencing lentiviruses. Cells were injected at four different doses, with *n* = 10 mice per dose. Tumor incidence at each cell dose was used to infer CSC frequency via ELDA. Data are presented as mean ± SD unless otherwise indicated. Two‐group comparisons were performed using a two‐tailed unpaired Student's *t*‐test. For comparisons among multiple groups, one‐way ANOVA with post hoc multiple‐comparisons testing was used. OE=overexpression. ^**^ *p* < 0.01, ^***^ *p* < 0.001.

Building on these findings, we next investigated whether OCIAD2 contributes to cisplatin response by promoting stemness in HNSCC. We directly assessed OCIAD2 expression in freshly resected tumor tissues from PDX models. Both qRT‐PCR and immunoblotting revealed significantly elevated OCIAD2 levels in Cis‐R PDXs compared to Cis‐S counterparts (Figure 3A,B). To functionally validate this observation, OCIAD2 expression was modulated in primary tumor cells derived from the PDX#4 and PDX#5 models. These primary cells displayed an epithelial marker profile, characterized by expression of CK14 and EpCAM and absence of the fibroblast marker α‐SMA, confirming their epithelial origin (Figure S1M). OCIAD2 overexpression markedly upregulated canonical CSC markers, including CD133, CD44, SOX2, and BMI1 (Figure 3C). In vivo limiting dilution assays further demonstrated that OCIAD2 overexpression enhanced tumor‐initiating capacity and increased CSC frequency (Figure 3D–F). Conversely, OCIAD2 silencing in Cis‐R PDX#5 cells suppressed stemness‐related protein expression and reduced in vivo tumor initiation (Figure 3G–J). These results underscore the role of OCIAD2 as a key mediator linking stem‐like traits to altered chemotherapy response in HNSCC.

### 3.3 OCIAD2 Modulates Cisplatin Response and Stem‐Like Traits in HNSCC through Activation of Integrin–FAK–PI3K–AKT–mTOR Signaling

**FIGURE 4:**
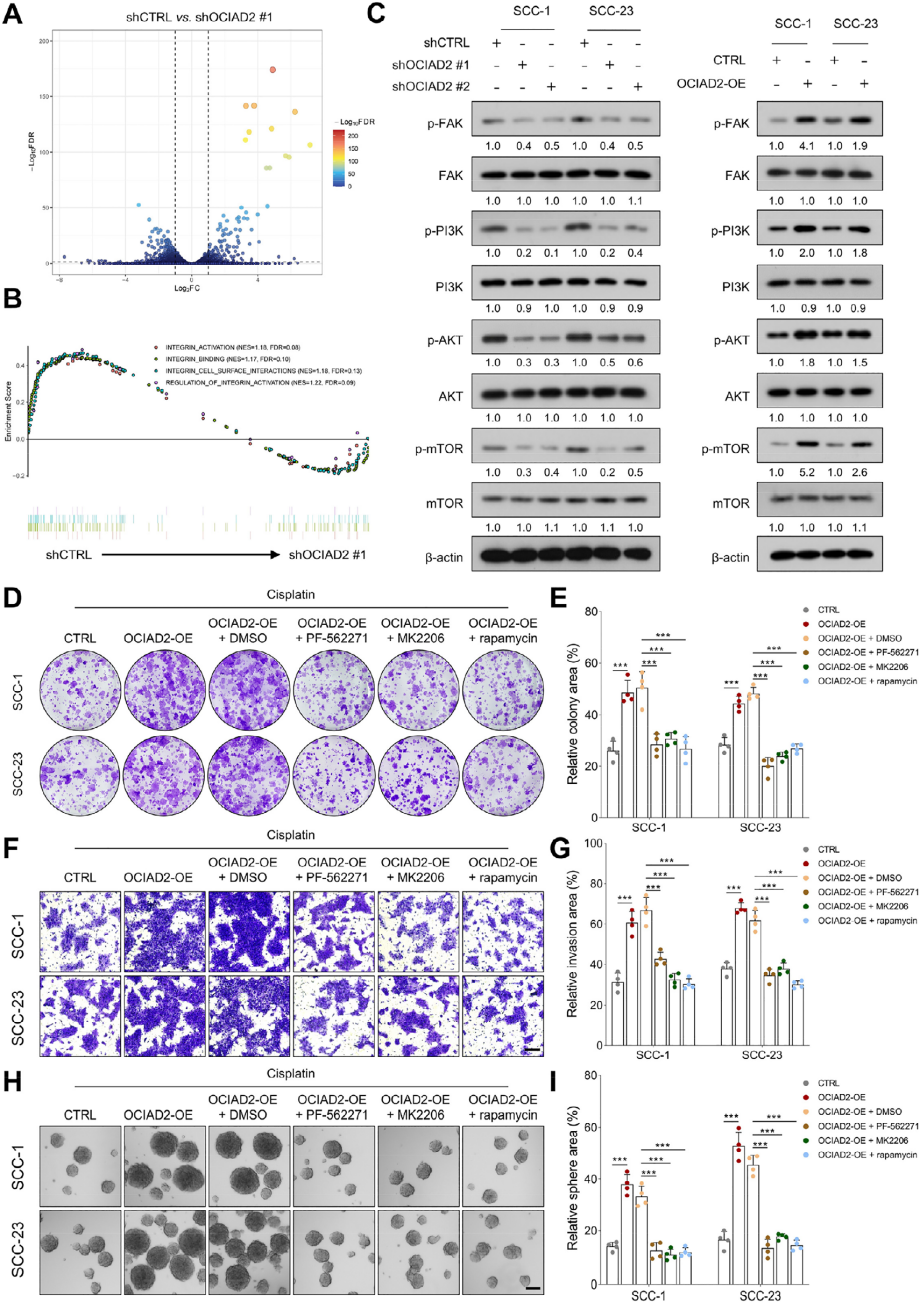
OCIAD2 activates the integrin–FAK–PI3K–AKT–mTOR signaling pathway in HNSCC cells. (A) RNA sequencing–based differential expression analysis in SCC‐1 cells with OCIAD2 knockdown, presented as a volcano plot (*n* = 3). (B) GSEA showing significant enrichment of integrin signaling‐related gene signatures in control SCC‐1 cells compared to OCIAD2‐depleted cells. (C) Immunoblot analysis of phosphorylated and total FAK, PI3K, AKT, and mTOR in SCC‐1 and SCC‐23 cells following OCIAD2 knockdown or overexpression (*n* = 3). (D,E) Colony formation assays in OCIAD2‐overexpressing SCC‐1 and SCC‐23 cells treated with cisplatin, in the presence of DMSO, PF‐562271 (FAK inhibitor), MK2206 (AKT inhibitor), or rapamycin (mTOR inhibitor) (*n* = 4). (F,G) Transwell invasion assays in SCC‐1 and SCC‐23 cells overexpressing OCIAD2 and treated with cisplatin in the presence of DMSO, PF‐562271, MK2206, or rapamycin (*n* = 4). Scale bar, 100 µm. (H,I) Tumorsphere formation assays were conducted under the same cellular and pharmacological conditions (*n* = 4). Scale bar, 100 µm. Data are presented as mean ± SD unless otherwise indicated. For comparisons among multiple groups, one‐way ANOVA with post hoc multiple‐comparisons testing was used. OE=overexpression. ^***^ *p* < 0.001.

To elucidate the molecular basis through which OCIAD2 regulates cisplatin response and stem‐like traits in HNSCC, we performed RNA sequencing of SCC‐1 cells following lentiviral knockdown of OCIAD2. Gene set enrichment analysis (GSEA) revealed a significant negative enrichment of integrin‐related signaling signatures upon OCIAD2 depletion, suggesting a mechanistic link between OCIAD2 and integrin pathway activation (Figure 4A,B). Integrin signaling is known to activate FAK, which in turn triggers the PI3K–AKT–mTOR cascade—a central axis implicated in promoting tumor survival, therapeutic resistance, and malignant progression in HNSCC [18, 19, 20]. Immunoblotting confirmed that OCIAD2 overexpression enhanced phosphorylation of FAK, PI3K, AKT, and mTOR, whereas OCIAD2 silencing attenuated their phosphorylation without affecting total protein levels (Figure 4C).

To investigate whether OCIAD2 modulates cisplatin response and tumor aggressiveness via the integrin–FAK–PI3K–AKT–mTOR signaling cascade, we performed pathway inhibition experiments. Under cisplatin treatment, OCIAD2 overexpression significantly increased colony size and number in SCC‐1 and SCC‐23 cells, whereas inhibition of FAK (PF‐562271), PI3K (MK2206), or mTOR (Rapamycin) abolished these effects (Figure 4D,E). Consistently, OCIAD2‐driven increases in cell migration, invasion, and tumorsphere formation were effectively suppressed by these inhibitors (Figure 4F–I). At the molecular level, OCIAD2 overexpression elevated PCNA and key stemness markers such as SOX2, CD44, and CD133, while pathway inhibition reversed these changes. Moreover, OCIAD2‐induced downregulation of E‐cadherin and upregulation of N‐cadherin were also reversed upon pharmacological blockade of the pathway (Figure S2A,B). Together, these findings position OCIAD2 as an upstream modulator of integrin–FAK–PI3K–AKT–mTOR signaling, thereby influencing cisplatin response and tumor progression in HNSCC.

To further evaluate the involvement of AKT signaling in OCIAD2‐mediated oncogenic behaviors, we performed genetic rescue experiments using a constitutively active form of AKT (myristoylated AKT, Myr‐AKT) in OCIAD2‐silenced cells. OCIAD2 knockdown reduced AKT phosphorylation at Ser473 and was accompanied by decreased expression of stemness‐associated markers, including SOX2, CD44, BMI1, and CD133, at both the protein and mRNA levels, in both SCC‐1 and SCC‐23 cells. Reactivation of AKT signaling by Myr‐AKT expression partially restored AKT phosphorylation and the expression of these markers (Figure S3A,B). Consistent with these molecular changes, OCIAD2 depletion significantly impaired clonogenic growth and tumorsphere formation under cisplatin treatment, whereas enforced AKT activation alleviated these defects in both cell lines (Figure S3C–F). Together, these data support a functional contribution of AKT signaling to OCIAD2‐dependent regulation of oncogenic behaviors and cisplatin response in HNSCC.

### 3.4 OCIAD2 Physically Associates with Integrin β1 in HNSCC

**FIGURE 5:**
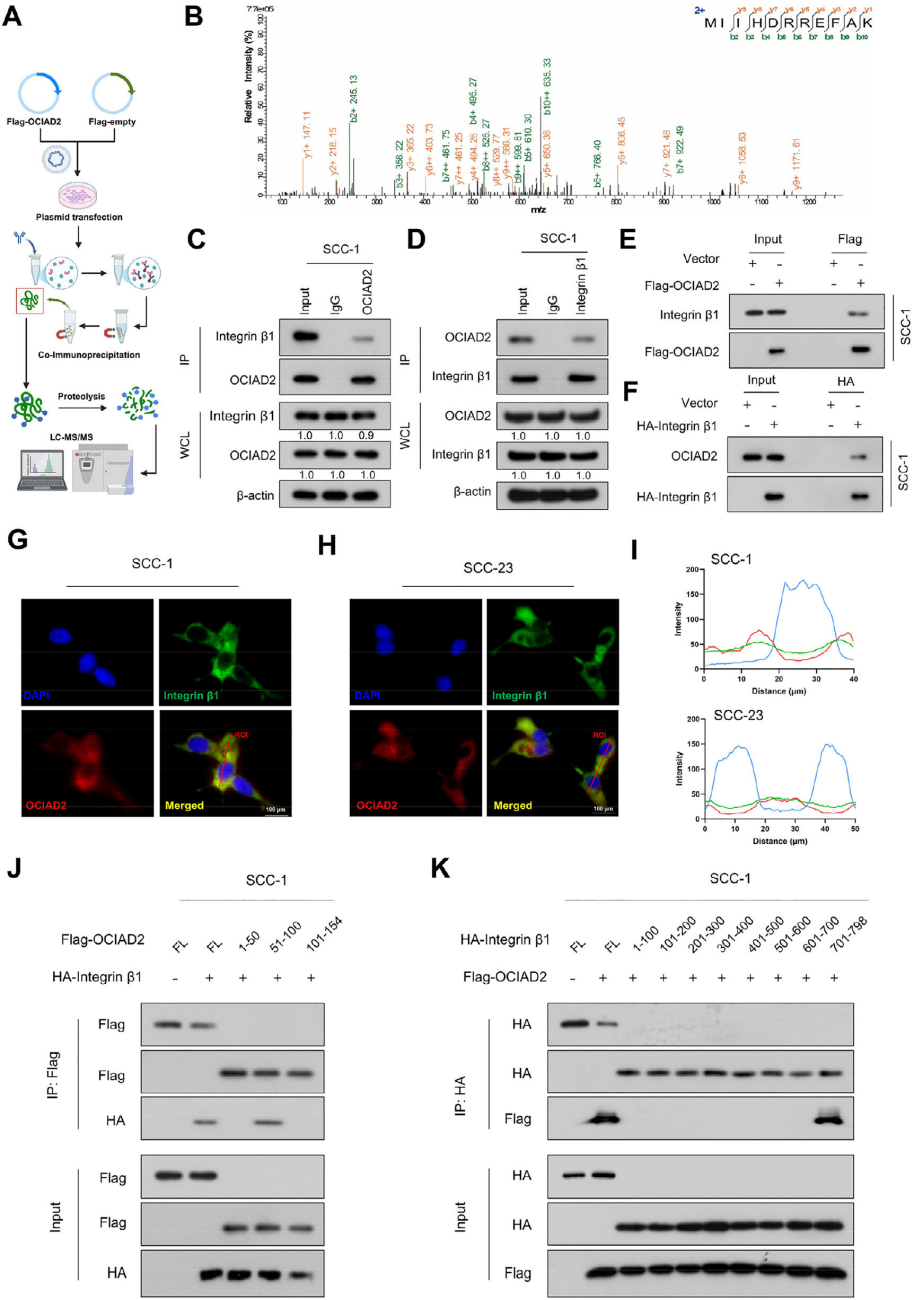
OCIAD2 directly interacts with integrin β1 in HNSCC cells. (A) Schematic illustration of the experimental workflow for identifying OCIAD2‐interacting proteins using Co‐IP followed by LC–MS/MS. (B) Representative mass spectrometry spectrum identifying integrin β1 as a potential OCIAD2‐interacting partner. (C,D) Co‐IP assays demonstrating the interaction between endogenous OCIAD2 and integrin β1 in SCC‐1 cells using anti‐OCIAD2 or anti‐integrin β1 antibodies (*n* = 3). (E,F) Co‐IP of exogenously expressed Flag–OCIAD2 and HA–integrin β1 in SCC‐1 cells using anti‐Flag or anti‐HA antibodies (*n* = 3). (G–I) Immunofluorescence staining of OCIAD2 and integrin β1 in SCC‐1 and SCC‐23 cells; colocalization was assessed by fluorescence intensity line scan analysis (*n* = 3). (J) Domain mapping of OCIAD2 using Flag‐tagged truncation mutants co‐expressed with full‐length HA–integrin β1 to define the region required for binding (*n* = 3). (K) Reciprocal domain mapping of integrin β1 using HA‐tagged truncation constructs co‐expressed with full‐length Flag–OCIAD2. Protein interactions were validated by Co‐IP and immunoblotting (*n* = 3).

To further define the mechanistic basis by which OCIAD2 engages the integrin signaling axis, we investigated whether OCIAD2 physically associates with upstream components of the pathway. To explore this possibility, we performed Co‐IP followed by mass spectrometry in SCC‐1 cells ectopically expressing Flag‐tagged OCIAD2 (Figure 5A). Integrin β1 was identified as a candidate binding partner (Figure 5B; Table S3). Co‐IP assays confirmed the interaction between endogenous OCIAD2 and integrin β1, which was further validated using epitope‐tagged constructs in overexpression systems (Figure 5C–F; Figure S4A–D).

Immunofluorescence staining revealed a clear colocalization of OCIAD2 and integrin β1 in SCC‐1 and SCC‐23 cells, as further confirmed by line scan analysis showing overlapping fluorescence intensity profiles (Figure 5G–I). To identify the specific regions mediating the interaction between OCIAD2 and integrin β1, we performed Co‐IP assays using a panel of truncation mutants. In SCC‐1 cells co‐transfected with HA‐tagged integrin β1 and various Flag‐tagged OCIAD2 fragments, only the 51–100 amino acid segment retained binding ability, whereas the 1–50 and 101–154 fragments failed to interact with integrin β1 (Figure 5J), indicating that this central region is essential for the association. Reciprocal mapping using integrin β1 truncations revealed that OCIAD2 specifically bound to the 701–798 region, corresponding to the cytoplasmic tail of integrin β1 (Figure 5K). These results define the minimal sequence regions required for their interaction and provide mechanistic insight into how OCIAD2 engages the integrin signaling pathway.

### 3.5 OCIAD2 Promotes its Downstream Effects through Post‐Transcriptional Stabilization of Integrin β1

**FIGURE 6:**
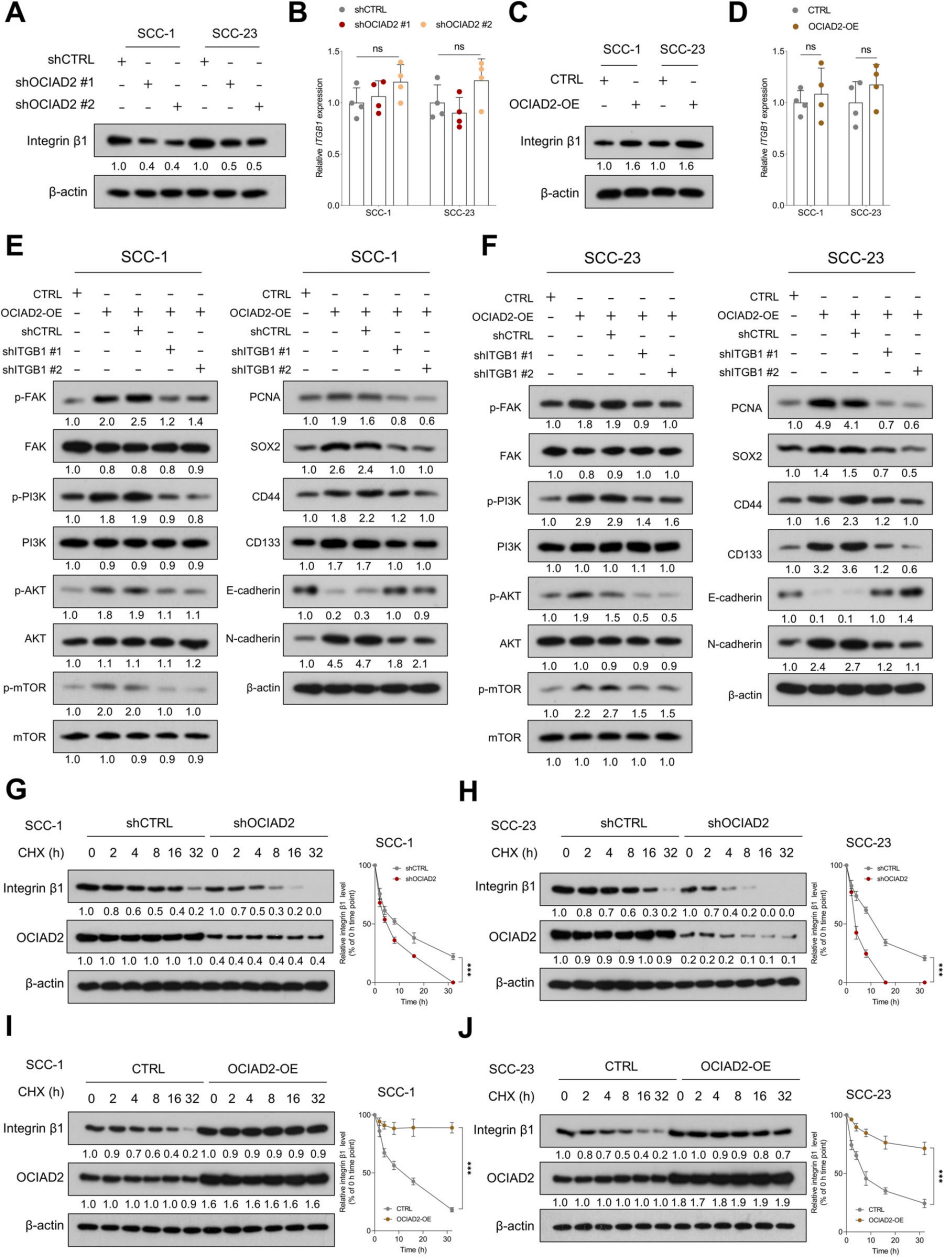
OCIAD2 sustains integrin β1 protein stability and promotes integrin β1–dependent signaling in HNSCC cells. (A,B) Immunoblot (*n* = 3) and qRT‐PCR (*n* = 4) analyses of integrin β1 expression in SCC‐1 and SCC‐23 cells following OCIAD2 knockdown. (C,D) Immunoblot (*n* = 3) and qRT‐PCR (*n* = 4) analyses of integrin β1 levels in SCC‐1 and SCC‐23 cells overexpressing OCIAD2. (E,F) Immunoblot analysis of phosphorylated and total FAK, PI3K, AKT, and mTOR, along with PCNA, SOX2, CD44, CD133, E‐cadherin, and N‐cadherin in OCIAD2‐overexpressing SCC‐1 and SCC‐23 cells, with or without integrin β1 knockdown (*n* = 3). (G,H) CHX chase assays in SCC‐1 and SCC‐23 cells with OCIAD2 knockdown to assess integrin β1 protein stability (*n* = 4). (I,J) CHX chase assays in SCC‐1 and SCC‐23 cells following OCIAD2 overexpression to evaluate integrin β1 degradation dynamics (*n* = 4). Data are presented as mean ± SD unless otherwise indicated. Two‐group comparisons were performed using a two‐tailed unpaired Student's *t*‐test. For comparisons among multiple groups, one‐way ANOVA with post hoc multiple‐comparisons testing was used. OE=overexpression. ^***^ *p* < 0.001; ns, not significant.

Given the physical interaction between OCIAD2 and integrin β1, we next examined whether OCIAD2 regulates integrin β1 expression. Immunoblotting revealed that OCIAD2 depletion led to a marked reduction in integrin β1 protein levels in both SCC‐1 and SCC‐23 cells, while quantitative PCR showed no significant changes in *ITGB1* mRNA expression (Figure 6A,B). Conversely, ectopic expression of OCIAD2 increased integrin β1 protein abundance without affecting transcript levels (Figure 6C–D), suggesting a post‐transcriptional mode of regulation.

To determine whether integrin β1 mediates the pro‐tumorigenic effects of OCIAD2, we conducted rescue experiments in OCIAD2‐overexpressing cells. Functional assays revealed that OCIAD2 overexpression markedly enhanced colony formation, invasive capacity, and tumorsphere growth under cisplatin treatment, whereas integrin β1 knockdown abolished these phenotypes (Figure S5A–F). At the molecular level, suppression of integrin β1 abrogated OCIAD2‐induced activation of the FAK–PI3K–AKT–mTOR signaling cascade, as evidenced by reduced phosphorylation of key signaling components. Moreover, integrin β1 silencing reversed the upregulation of PCNA and stemness‐associated markers (SOX2, CD44, and CD133), and restored E‐cadherin while reducing N‐cadherin (Figure 6E,F). To further investigate the mechanism by which OCIAD2 regulates integrin β1 protein levels, we conducted CHX chase assays to evaluate integrin β1 stability. OCIAD2 knockdown markedly reduced the half‐life of integrin β1, whereas OCIAD2 overexpression prolonged its stability and delayed protein degradation in both SCC‐1 and SCC‐23 cells (Figure 6G–J). These results indicate that OCIAD2 enhances the stability of integrin β1 protein, thereby sustaining downstream oncogenic signaling and reinforcing the chemoresistant and stem‐like phenotypes of HNSCC cells.

### 3.6 OCIAD2 Maintains Integrin β1 Membrane Localization by Preventing Lysosomal Degradation and Promoting Recycling into Lipid Raft Microdomains

**FIGURE 7:**
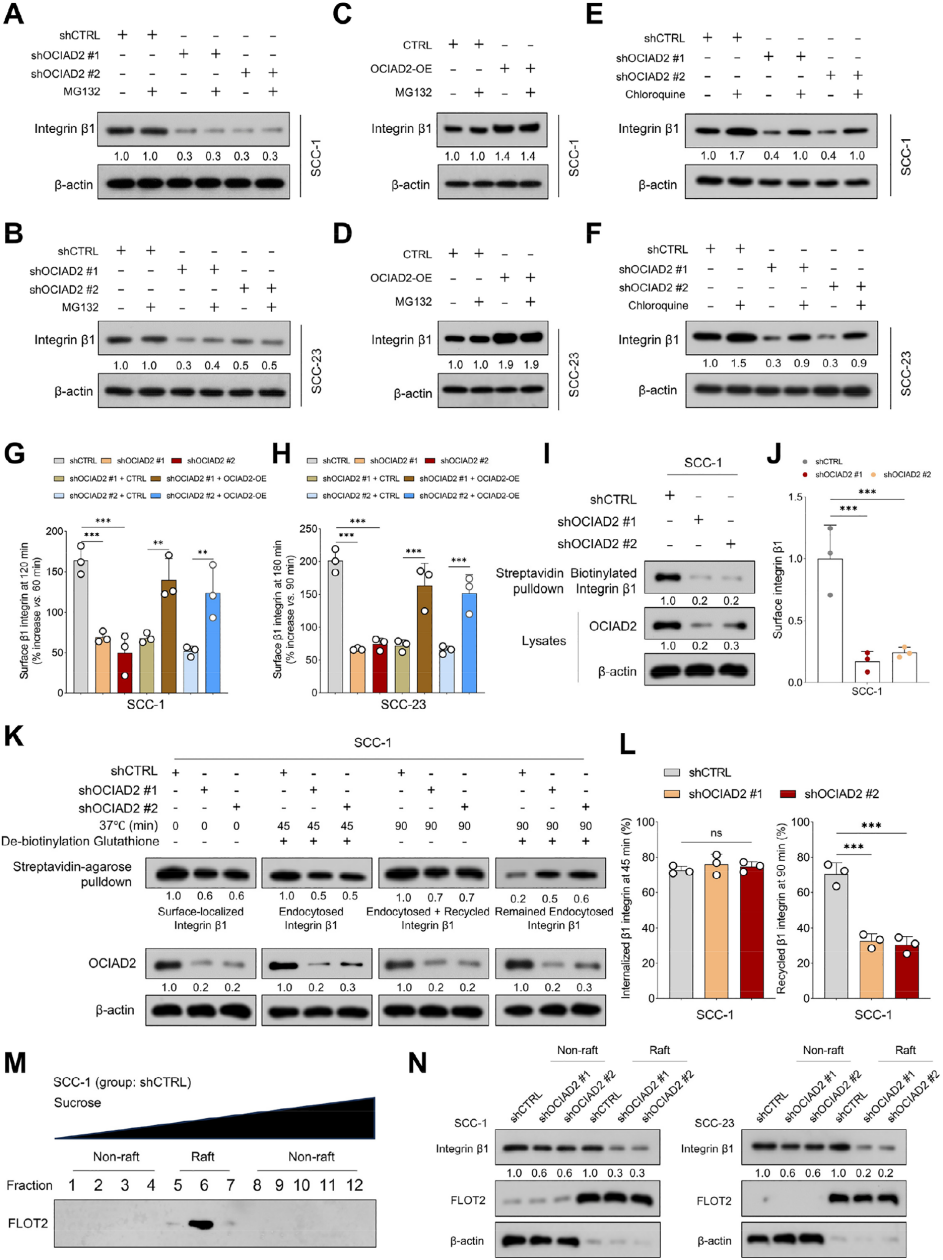
OCIAD2 promotes integrin β1 recycling to lipid rafts and prevents lysosomal degradation in HNSCC cells. (A–D) Immunoblot analysis of integrin β1 protein levels in SCC‐1 and SCC‐23 cells upon OCIAD2 knockdown or overexpression, with or without MG132 treatment (*n* = 3). (E,F) Immunoblot analysis of integrin β1 levels in OCIAD2‐depleted SCC‐1 and SCC‐23 cells treated with chloroquine (*n* = 3). (G,H) Surface expression of integrin β1 at a later time point after NZ washout relative to an earlier time point in SCC‐1 and SCC‐23 cells transduced with either shCTRL or shOCIAD2, with or without OCIAD2 rescue (*n* = 3). (I,J) Detection of surface integrin β1 in SCC‐1 cells with or without OCIAD2 knockdown using biotinylation‐based streptavidin pulldown and immunoblotting (*n* = 3). (K,L) Biotinylation‐based recycling assays in SCC‐1 cells assessing surface, internalized, recycled, and retained integrin β1 following OCIAD2 knockdown (*n* = 3). (M) Immunoblot analysis of FLOT2 distribution across a sucrose density gradient in SCC‐1 and SCC‐23 cells to define lipid raft–enriched membrane fractions. Cell lysates were subjected to sucrose gradient ultracentrifugation and fractionated sequentially into 12 fractions. Based on FLOT2 enrichment, fractions 5–7 were designated as lipid raft fractions, whereas fractions 1–4 and 8–12 were classified as non‐raft fractions (*n* = 3). (N) Subcellular fractionation analysis of integrin β1 distribution across all 12 sucrose gradient fractions in SCC‐1 and SCC‐23 cells expressing control or OCIAD2‐targeting shRNAs. Raft and non‐raft fractions were assigned according to the FLOT2‐defined fractionation shown in (M) (*n* = 3). Data are presented as mean ± SD unless otherwise indicated. Two‐group comparisons were performed using a two‐tailed unpaired Student's *t*‐test. For comparisons among multiple groups, one‐way ANOVA with post hoc multiple‐comparisons testing was used. OE=overexpression. ^***^ *p* < 0.001; ns, not significant.

To elucidate the mechanism by which OCIAD2 regulates integrin β1 protein stability, we examined whether proteasomal or lysosomal degradation pathways were involved. Treatment with the proteasome inhibitor MG132 failed to restore integrin β1 levels in OCIAD2‐knockdown SCC‐1 and SCC‐23 cells (Figure 7A–D), indicating that proteasome‐mediated degradation is not the predominant route of integrin β1 turnover in this context. In contrast, inhibition of lysosomal function with chloroquine significantly rescued integrin β1 expression following OCIAD2 depletion (Figure 7E,F), suggesting that OCIAD2 maintains integrin β1 stability by preventing its lysosomal degradation.

Given that lysosomal degradation is tightly coupled to membrane trafficking events, we next asked whether OCIAD2 influences integrin β1 endocytic recycling [21, 22]. To address this, we employed a nocodazole (NZ) washout assay to assess membrane trafficking dynamics. Flow cytometric analysis showed that OCIAD2 knockdown impaired the restoration of surface integrin β1 levels after NZ removal, while OCIAD2 overexpression enhanced recycling efficiency (Figure 7G,H; Figure S6A,B). Consistently, surface biotinylation assays demonstrated a substantial decrease in membrane‐localized integrin β1 following OCIAD2 depletion (Figure 7I,J; Figure S6C,D). To further dissect the impact of OCIAD2 on integrin β1 membrane dynamics, we performed a biotin‐based endocytosis and recycling assay. While OCIAD2 knockdown did not significantly affect the internalization of integrin β1, it markedly impaired the recycling of internalized integrin β1 back to the plasma membrane (Figure 7K,L; Figure S6E,F). These findings demonstrate that OCIAD2 is dispensable for integrin β1 endocytosis but is required for its efficient recycling.

Notably, lipid raft microdomains serve as critical regulatory platforms for integrin family proteins, orchestrating integrin‐mediated cell adhesion and signal transduction through spatial compartmentalization and protein clustering [23, 24, 25]. To further investigate whether OCIAD2 influences the subcellular distribution of integrin β1, we separated raft and non‐raft membrane fractions by sucrose gradient centrifugation (Figure 7M; Figure S7A,B). Western blot analysis showed that integrin β1 was present in both raft and non‐raft fractions under control conditions. However, OCIAD2 knockdown preferentially reduced integrin β1 abundance in raft fractions, with a less pronounced effect in non‐raft fractions (Figure 7N), indicating that OCIAD2 is critical for maintaining the lipid raft localization of integrin β1.

To determine whether lipid raft integrity is required for OCIAD2‐mediated signaling and oncogenic behaviors, we disrupted membrane lipid rafts using methyl‐β‐cyclodextrin (MβCD) and assessed downstream molecular and functional effects. In both SCC‐1 and SCC‐23 cells, OCIAD2 overexpression enhanced integrin–FAK–PI3K–AKT signaling, whereas MβCD markedly attenuated pathway activation (Figure S8A,B). Consistent with this effect, MβCD treatment suppressed OCIAD2‐induced upregulation of PCNA and stemness‐associated markers, including SOX2, CD44, and CD133. Functionally, OCIAD2 overexpression significantly promoted clonogenic growth and tumorsphere formation, while pharmacological disruption of lipid rafts largely abolished these effects in both cell lines (Figure S8C–F).

### 3.7 SNX17 Mediates OCIAD2‐Dependent Recycling and Membrane Localization of Integrin β1 to Sustain Oncogenic Signaling

**FIGURE 8:**
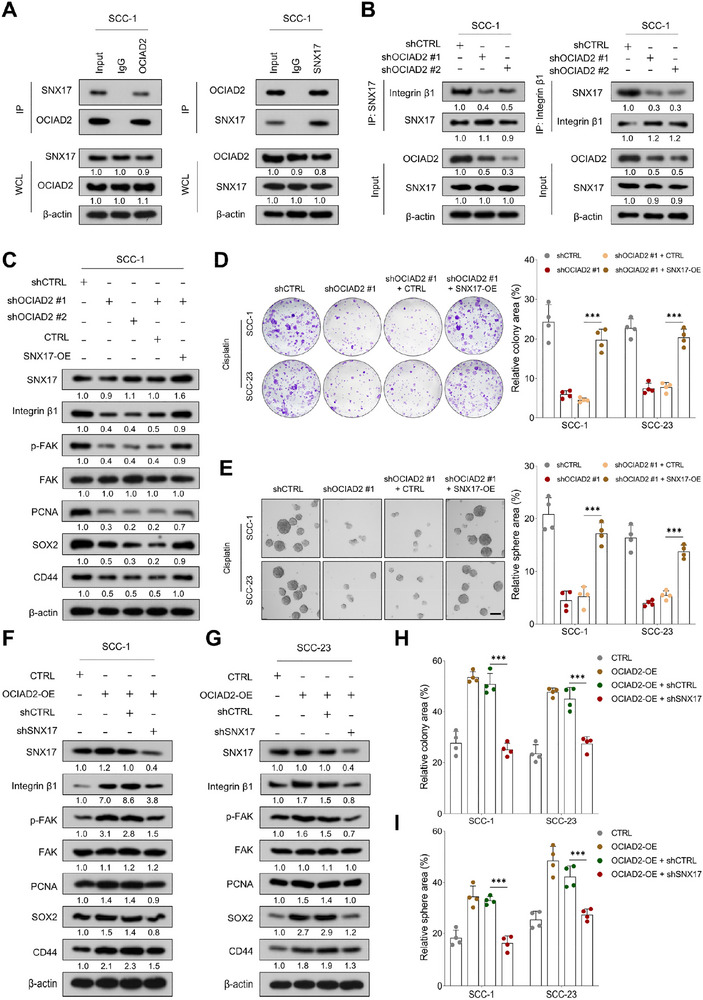
SNX17 mediates OCIAD2‐driven recycling of integrin β1 to sustain oncogenic signaling. (A) Co‐IP assays showing the interaction between OCIAD2 and SNX17 in SCC‐1 cells using anti‐OCIAD2 or anti‐SNX17 antibodies (*n* = 3). (B) Co‐IP of endogenous SNX17 with integrin β1 in SCC‐1 cells upon OCIAD2 knockdown (*n* = 3). (C) Immunoblot analysis of integrin β1, p‐FAK, FAK, PCNA, SOX2, and CD44 in SCC‐1 cells transduced with control or OCIAD2‐targeting shRNAs, with or without SNX17 overexpression (*n* = 3). (D,E) Colony formation and tumorsphere formation assays in SCC‐1 and SCC‐23 cells subjected to OCIAD2 knockdown, with or without rescue by SNX17 overexpression (*n* = 4). Scale bar, 100 µm. (F,G) Immunoblot analysis of the indicated proteins in SCC‐1 and SCC‐23 cells with OCIAD2 overexpression, in the presence or absence of SNX17 knockdown (*n* = 3). (H,I) Colony formation and tumorsphere formation assays were performed in SCC‐1 and SCC‐23 cells overexpressing OCIAD2, with or without concurrent SNX17 depletion (*n* = 4). Data are presented as mean ± SD unless otherwise indicated. For comparisons among multiple groups, one‐way ANOVA with post hoc multiple‐comparisons testing was used. OE=overexpression. ^***^ *p* < 0.001; ns, not significant.

To investigate the mechanism by which OCIAD2 regulates integrin β1 recycling, we examined the involvement of sorting nexin 17 (SNX17), a FERM‐like domain–containing protein known to bind the NPXY motif of β integrins and facilitate their endosome‐to‐plasma membrane recycling [26, 27]. Co‐IP assays revealed a specific interaction between endogenous OCIAD2 and SNX17 in HNSCC cells (Figure 8A; Figure S9A,B). Notably, knockdown of OCIAD2 substantially weakened the association between SNX17 and integrin β1 (Figure 8B; Figure S9C,D).

To assess whether SNX17 can compensate for the loss of OCIAD2, we ectopically expressed SNX17 in OCIAD2‐deficient cells. Western blot analysis showed that SNX17 overexpression partially restored the reduced levels of integrin β1 and its downstream signaling components, including phosphorylated FAK, PCNA, SOX2, and CD44, following OCIAD2 silencing (Figure 8C; Figure S9E). Consistently, enforced SNX17 expression alleviated the impairment in colony formation and tumorsphere growth observed in OCIAD2‐deficient cells under cisplatin treatment (Figure 8D,E). We next examined whether SNX17 contributes to OCIAD2‐driven oncogenic phenotypes. Although OCIAD2 overexpression enhanced integrin β1–associated signaling and malignant behaviors, depletion of SNX17 attenuated these effects, leading to reduced integrin β1 expression and suppression of OCIAD2‐induced colony formation and tumorsphere growth (Figure 8F–I). In addition, SNX17 overexpression partially mitigated the reduction of integrin β1 within lipid raft fractions caused by OCIAD2 depletion (Figure S9F,G). Taken together, these findings suggest that SNX17 supports OCIAD2‐mediated integrin β1 recycling, membrane retention, and downstream oncogenic signaling, rather than acting as an exclusive or sole determinant of these processes.

### 3.8 Targeting the OCIAD2–SNX17–integrin β1 Axis Suppresses Tumor Growth and Enhances Cisplatin Sensitivity In Vivo

**FIGURE 9:**
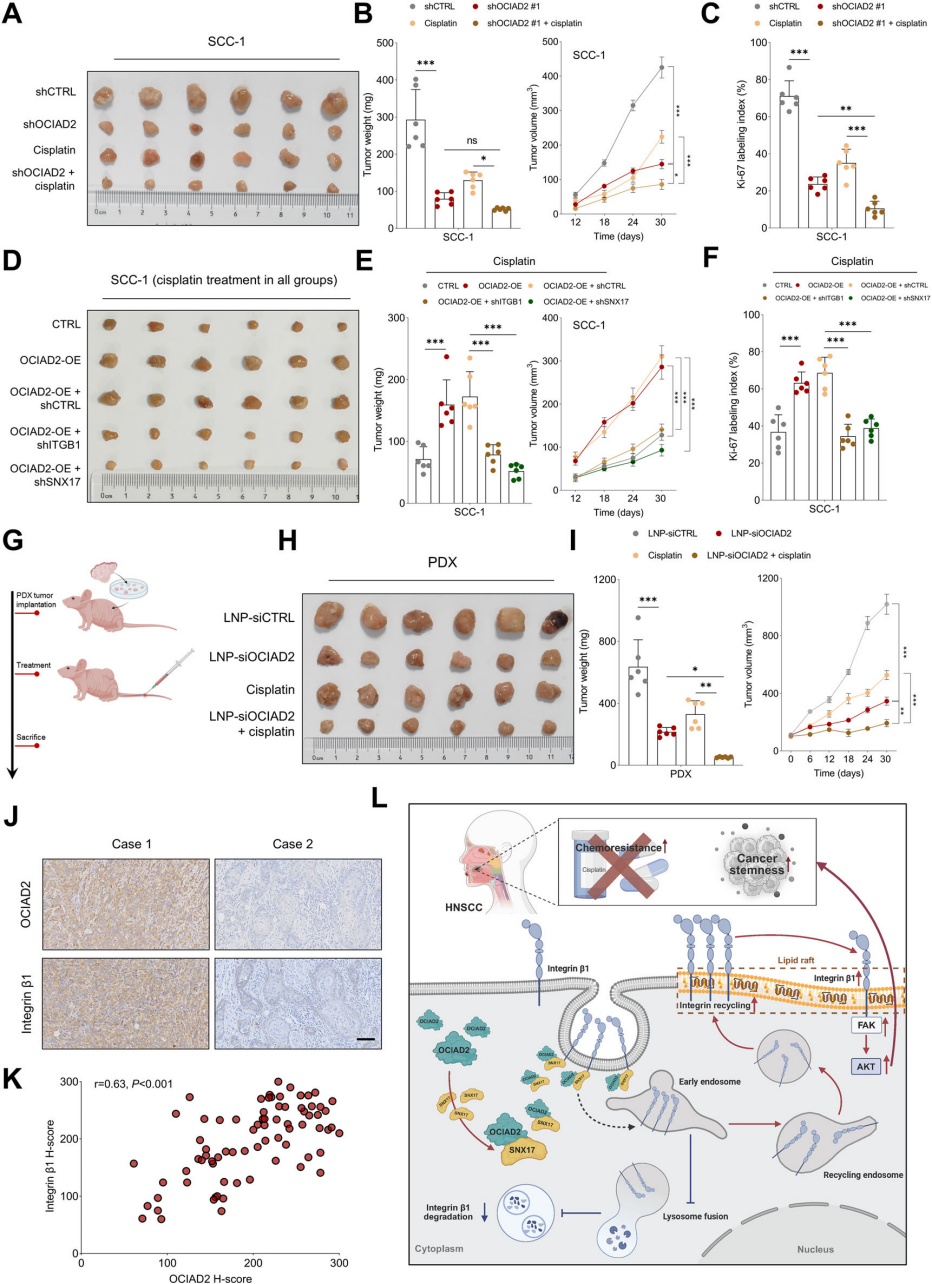
Targeting OCIAD2 enhances cisplatin sensitivity in HNSCC xenograft and PDX models. (A–C) Tumor burden and Ki‐67 staining in xenografts derived from SCC‐1 cells expressing control or OCIAD2‐targeting shRNAs under cisplatin or vehicle treatment (four groups, *n* = 6 mice per group). (D–F) Tumor size, weight, and Ki‐67 staining in xenografts derived from SCC‐1 cells overexpressing OCIAD2 with or without integrin β1 or SNX17 knockdown, all subjected to cisplatin treatment (five groups, *n* = 6 mice per group). (G–I) Tumor growth analysis of PDX models receiving liposomal nanoparticles loaded with control or OCIAD2‐targeting siRNA, administered alone or in combination with cisplatin. Tumor burden was assessed by measuring tumor weight and volume over time (four groups, *n* = 6 mice per group). (J,K) Spearman correlation analysis of OCIAD2 and integrin β1 immunohistochemical staining intensities in primary HNSCC tissues from the in‐house cohort (*n* = 76). (L) Schematic model of OCIAD2 promoting SNX17‐dependent integrin β1 recycling to lipid rafts, sustaining downstream signaling and enhancing cisplatin resistance. Created with BioRender.com. Data are presented as mean ± SD unless otherwise indicated. For comparisons among multiple groups, one‐way ANOVA with post hoc multiple‐comparisons testing was used. OE=overexpression, LNP=lipid nanoparticles. ^*^ *p* < 0.05, ^**^ *p* < 0.01 and ^***^ *p* < 0.001; ns, not significant.

To evaluate the functional relevance of OCIAD2 in mediating tumor growth and chemoresistance, we conducted in vivo studies using HNSCC xenograft and PDX models. In SCC‐1 xenografts, OCIAD2 silencing significantly suppressed tumor growth, as reflected by reduced tumor volume and delayed growth kinetics. While cisplatin treatment alone also inhibited tumor progression, the combination of OCIAD2 depletion with cisplatin further attenuated tumor growth over time, despite no significant difference in endpoint tumor weight between the shOCIAD2 group and the shOCIAD2 plus cisplatin group (Figure 9A,B). Consistently, Ki‐67 immunostaining revealed a reduction in proliferative cells following the combined therapy (Figure 9C; Figure S10A). To assess the contribution of integrin β1 and SNX17 to OCIAD2‐driven tumorigenesis, SCC‐1 xenografts overexpressing OCIAD2 were subjected to ITGB1 or SNX17 knockdown. Despite enforced OCIAD2 expression, silencing either ITGB1 or SNX17 significantly diminished tumor growth under cisplatin treatment, as evidenced by reductions in tumor burden and Ki‐67 positivity (Figure 9D–F; Figure S10B). Similar trends were observed in HPV‐positive SCC‐23 xenografts, where OCIAD2 depletion suppressed tumor growth, and the combination with cisplatin resulted in a greater reduction in tumor burden and slower tumor growth compared with single treatments (Figure S11A,B).

To evaluate the translational potential of targeting OCIAD2, we employed a PDX model treated with lipid nanoparticle–encapsulated OCIAD2 siRNA (LNP‐siOCIAD2). While either LNP‐siOCIAD2 or cisplatin monotherapy reduced tumor burden, the combination induced a significantly enhanced therapeutic response (Figure 9G–I). Importantly, LNP‐siOCIAD2 administration was well tolerated in vivo. No significant differences were observed in body weight, serum liver enzymes (ALT and AST), or renal function indicators (BUN and creatinine) among treatment groups (Figure S12A–E). Histological examination of major organs, including the heart, liver, spleen, lung, and kidney, revealed no overt pathological abnormalities following LNP‐siOCIAD2 treatment (Figure S12F), indicating a favorable safety profile.

To further explore the clinical relevance of this pathway, immunohistochemical analysis of an in‐house HNSCC tissue microarray demonstrated a significant positive correlation between OCIAD2 and integrin β1 expression levels (Figure 9J,K), supporting the clinical validity of our mechanistic findings.

## 4 Discussion

While cisplatin‐based chemotherapy remains a principal therapeutic option in the management of HNSCC, its long‐term benefit is often undermined by the emergence of drug resistance [28]. Rather than resulting solely from genetic mutations, resistance frequently reflects dynamic cellular adaptations—such as increased plasticity, evasion of apoptosis, and reprogramming of signaling networks—that allow tumor cells to persist under therapeutic pressure [29, 30, 31]. In this context, the present study uncovers a previously uncharacterized role for OCIAD2 as a central mediator of chemoresistance and tumor progression in HNSCC, operating through the stabilization and endosomal recycling of integrin β1. By integrating transcriptomic analyses, mechanistic dissection of protein trafficking, and preclinical therapeutic validation, our findings reveal a critical OCIAD2–SNX17–integrin β1 axis that governs key malignant phenotypes, including stemness maintenance, survival under chemotherapeutic pressure, and sustained oncogenic signaling (Figure 9L).

OCIAD2 is a mitochondrial‐associated protein originally identified in the context of ovarian carcinoma, yet its functional roles across cancer types remain poorly defined [32, 33, 34]. Emerging studies have linked OCIAD2 to tumor progression, but its precise molecular functions and mechanistic contributions have largely remained elusive [14, 35]. Here, we identify OCIAD2 as a key regulator of cisplatin response in HNSCC, supported by converging evidence from patient tumor samples, mechanistic in vitro assays, and functional validation in PDX models. In particular, OCIAD2 silencing sensitized resistant tumors to cisplatin, and LNP‐formulated siOCIAD2 delivered significant antitumor efficacy in vivo, supporting its translational potential. Despite these encouraging findings, the clinical druggability of OCIAD2 warrants careful consideration. OCIAD2 lacks characterized enzymatic domains or clearly defined small‐molecule binding pockets, which poses a significant barrier to conventional inhibitor development. Accordingly, OCIAD2 may currently be considered a challenging—or “undruggable”—target using traditional small‐molecule approaches. However, this does not preclude the feasibility of alternative modalities. Recent advances in targeted protein degradation and RNA‐based therapeutics offer viable routes to therapeutically modulate OCIAD2, despite its undruggable structural features [36, 37].

While integrin β1 has long been implicated in tumor biology—supporting adhesion, migration, and activation of pro‐survival cascades such as the FAK–PI3K–AKT–mTOR pathway—its regulation at the post‐transcriptional and trafficking levels remains insufficiently understood [20, 38, 39, 40]. Our results demonstrate that OCIAD2 does not affect integrin β1 at the transcriptional level, but instead promotes its protein stability by preventing lysosome‐mediated degradation and enhancing its recycling to lipid raft domains. This spatial redistribution is not a passive event but a prerequisite for effective downstream signal propagation, as integrin clustering within raft microdomains amplifies focal adhesion signaling and anchors tumor‐promoting pathways [41]. The marked reduction of integrin β1 within raft fractions upon OCIAD2 silencing, despite only partial decreases in total integrin β1 levels, highlights the spatial sensitivity of integrin signaling and underscores the importance of membrane compartmentalization in tumor cell behavior.

Importantly, this spatial regulation is not merely a biochemical observation but has functional consequences for tumor behavior. Raft‐associated integrin β1 enhances signal fidelity and intensity by promoting integrin clustering, co‐receptor recruitment, and sustained activation of FAK and its downstream effectors. OCIAD2, by orchestrating this spatial enrichment, ensures persistent integrin signaling even under cisplatin‐induced stress, thereby supporting cancer cell survival and stem‐like phenotypes. Disruption of this trafficking route—either by silencing OCIAD2 or interfering with its downstream effector SNX17—attenuates the integrity of this signaling axis. These findings suggest that the spatial compartmentalization of integrin β1, governed by OCIAD2‐mediated recycling, may represent a key determinant of functional resistance to chemotherapy and offer a conceptual shift from traditional views that focus primarily on receptor expression or canonical downstream signaling.

Mechanistically, OCIAD2 emerges as a critical facilitator of SNX17‐mediated recycling. While SNX17 is known to recognize NPXY motifs in the cytoplasmic tails of β integrins and facilitate their endosome‐to‐membrane recycling, our data suggest that its activity is supported by the presence of OCIAD2 [26, 42]. Loss of OCIAD2 diminishes the SNX17–integrin β1 interaction, reduces surface integrin β1 levels, and disrupts its localization to lipid rafts, ultimately impairing FAK pathway activation. Conversely, enforced SNX17 expression can partially rescue integrin β1 expression and its raft association in OCIAD2‐deficient cells, suggesting a hierarchical relationship in which OCIAD2 stabilizes or scaffolds the recycling machinery.

This cooperative interaction likely reflects a multi‐layered regulatory model, in which OCIAD2 not only promotes the physical association between SNX17 and integrin β1, but may also influence the subcellular positioning or conformational availability of integrin β1 for retrieval. Given that endosomal trafficking is tightly coordinated by membrane curvature, lipid composition, and adaptor protein recruitment, OCIAD2 might function as a platform that spatially confines SNX17 activity to recycling‐competent compartments [43]. Alternatively, OCIAD2 may directly shield integrin β1 from misrouting into degradative pathways, thereby preserving a pool of signaling‐competent receptors for plasma membrane reinsertion.

Importantly, this interaction is not merely mechanistic but functionally consequential. Knockdown of SNX17 partially mirrors the functional consequences observed upon OCIAD2 depletion. These findings suggest that SNX17 acts downstream of OCIAD2 and contributes to OCIAD2‐associated oncogenic processes. From a translational perspective, the dual dependence of integrin β1 recycling on both OCIAD2 and SNX17 highlights the vulnerability of this axis. Therapeutic disruption at either node may destabilize the recycling circuit, weaken pro‐survival signaling, and resensitize resistant tumors to chemotherapy. As such, targeting the OCIAD2–SNX17–integrin β1 module may offer a focused strategy to impair chemoresistant cell states without broadly suppressing global integrin functions.

Beyond the experimental evidence derived from in vitro and in vivo models, our conclusions are reinforced by analyses of multiple independent public transcriptomic datasets. Across diverse GEO cohorts and the TCGA HNSCC dataset, OCIAD2 expression was reproducibly elevated in tumor tissues relative to normal or adjacent non‐tumor epithelium, with several datasets further revealing a progressive increase from normal epithelium through dysplasia to carcinoma. Notably, elevated OCIAD2 expression was consistently associated with unfavorable overall survival across independent patient cohorts. These large‐scale, clinically annotated datasets therefore provide robust, external support for our experimental findings and place OCIAD2 upregulation within a clinically relevant context. Together with the mechanistic data presented here, the convergence of experimental and evidence derived from independent patient datasets underscores OCIAD2 as a tumor‐associated factor with potential prognostic relevance in HNSCC.

While our study provides mechanistic and translational insights into OCIAD2‐driven integrin β1 recycling and cisplatin resistance in HNSCC, several limitations warrant consideration. First, although OCIAD2 is identified as a central regulator of integrin β1 stability and spatial organization, the upstream regulatory signals governing OCIAD2 expression and activation were not systematically examined. Given the dynamic nature of endosomal trafficking and therapy‐induced stress responses, OCIAD2 is likely integrated into broader regulatory programs that remain to be fully delineated. Second, the in vivo validation of OCIAD2 targeting was primarily performed in xenograft and patient‐derived xenograft models, which emphasize tumor cell–intrinsic mechanisms. These models do not fully capture the complexity of tumor–host interactions that may influence integrin‐dependent signaling and therapeutic response in clinical settings. Further extension into more physiologically representative systems may therefore help to refine the contextual scope of OCIAD2‐mediated trafficking in heterogeneous tumor environments.

## 5 Conclusion

This study identifies OCIAD2 as a central regulator of chemoresistance and tumor progression in HNSCC, acting through SNX17‐mediated recycling of integrin β1 to lipid raft microdomains. By sustaining integrin signaling under cisplatin‐induced stress, the OCIAD2–SNX17–integrin β1 axis supports malignant phenotypes and treatment resistance. Disrupting this pathway, particularly through RNA‐based strategies, markedly enhances cisplatin efficacy in preclinical models. These findings not only uncover a previously unrecognized trafficking‐dependent mechanism of drug resistance but also highlight OCIAD2 as a potential therapeutic target for sensitizing HNSCC to platinum‐based chemotherapy.

## Author Contributions

L.C., X.Y.Z., W.J.S., and S.S.S. conceived and designed the experiments. L.C., X.Y.Z., W.J.S., S.S.S., M.Y., P.L., M.Y.Z., Y.F.L., X.C., and B.G. wrote, reviewed, and revised the manuscript. L.C., X.Y.Z., W.J.S., M.Y., and S.S.S. developed the methodology. L.C., X.Y.Z., W.J.S., M.Y., and S.S.S. analyzed and interpreted the data. LC and XYZ supervised the study. All authors read and approved the final manuscript.

## Conflicts of Interest

The authors declare no conflicts of interest.

## Supporting information




**Supporting File**: advs74160‐sup‐0001‐SuppMat.pdf.

## Data Availability

The data that support the findings of this study are available on request from the corresponding author. The data are not publicly available due to privacy or ethical restrictions.
