# Supplementary material for: OCIAD2 Stabilizes Integrin β1 Signaling Through SNX17‐Mediated Endosomal Recycling to Lipid Rafts and Modulates Cisplatin Response in HNSCC
Source: Adv Sci (Weinh). 2026 Feb 8;13(20):e15452. doi: 10.1002/advs.202515452 (PMC13067770; doi:10.1002/advs.202515452)
Supplement: Supplementary file 1 — Supporting File: advs74160‐sup‐0001‐SuppMat.pdf. [file ADVS-13-e15452-s001.pdf]

OCIAD2 Stabilizes Integrin β1 Signaling Through SNX17-Mediated Endosomal Recycling to Lipid Rafts and Modulates Cisplatin Response in HNSCC

Li Cui[1, 2*, #], Shanshan Si[1*], Min Ye[1], Pei Lin[1], Meiyan Zou[1], Yunfan Lin[1], Xu Chen[1], Bing Guo[3], Wenjuan Sun[4#], Xinyuan Zhao[1#]

[1]Stomatological Hospital, School of Stomatology, Southern Medical University, Guangzhou, 510280, Guangdong, China.

[2]School of Dentistry, University of California, Los Angeles, Los Angeles, 90095, CA, USA.

[3]Department of Dentistry, The First Affiliated Hospital, Sun Yat-sen University, Guangzhou 510080, China.

[4]Department of Stomatology, The Third Affiliated Hospital, Sun Yat-sen University, Guangzhou, 510630, China.

*These authors contributed equally to this work.

[#]Correspondence

Li Cui, Email: licui@smu.edu.cn

Wenjuan Sun, Email: sunwj5@mail.sysu.edu.cn

Xinyuan Zhao, Email: zhaoxinyuan1989@smu.edu.cn

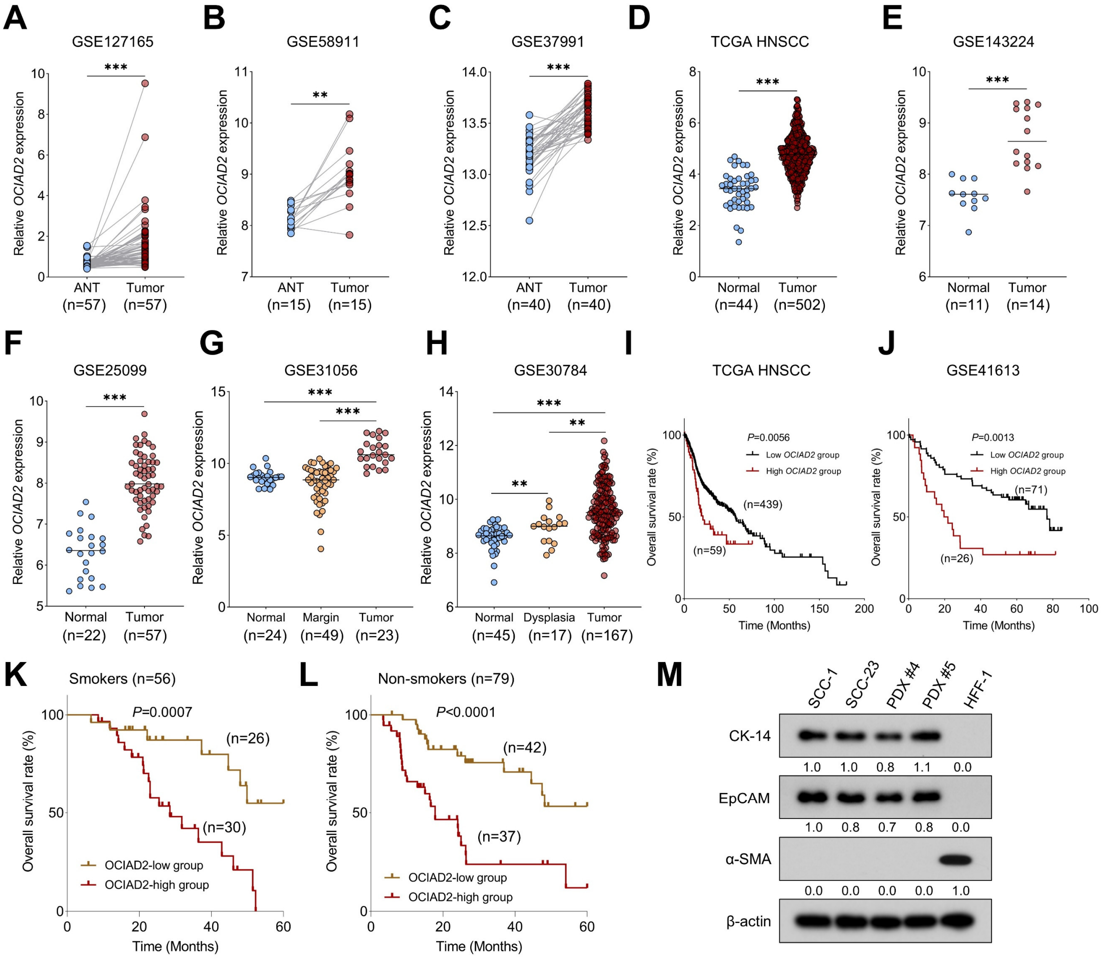

Supplementary Figure 1. *OCIAD2* is upregulated in HNSCC and predicts poor prognosis based on analyses of public transcriptomic datasets. (A–F) *OCIAD2* expression in tumor tissues compared to adjacent non-tumor (ANT) or normal tissues across GSE127165, GSE58911, GSE37991, TCGA-HNSCC, GSE143224, and GSE25099 datasets. (G) *OCIAD2* expression in normal, margin, and tumor tissues from the GSE31056 dataset. (H) *OCIAD2* expression in normal, dysplastic, and tumor tissues from the GSE30784 dataset. (I–J) Kaplan–Meier analysis of overall survival in HNSCC patients stratified by *OCIAD2* expression in TCGA-HNSCC and GSE41613 cohorts. (K–L) Kaplan–Meier analysis of overall survival in in-house HNSCC patients stratified by OCIAD2 expression within smokers (n=56) and non-smokers (n=79). Patients were divided into OCIAD2-high and OCIAD2-low groups based on the median expression value. Survival differences were assessed using the log-rank test. (M) Immunoblot analysis of epithelial (CK14, EpCAM) and mesenchymal (α-SMA) markers in SCC-1 and SCC-23 cell lines, PDX tumors, and human foreskin fibroblasts (HFF-1) (n=3). $^{**}P < 0.01$, $^{***}P < 0.001$.

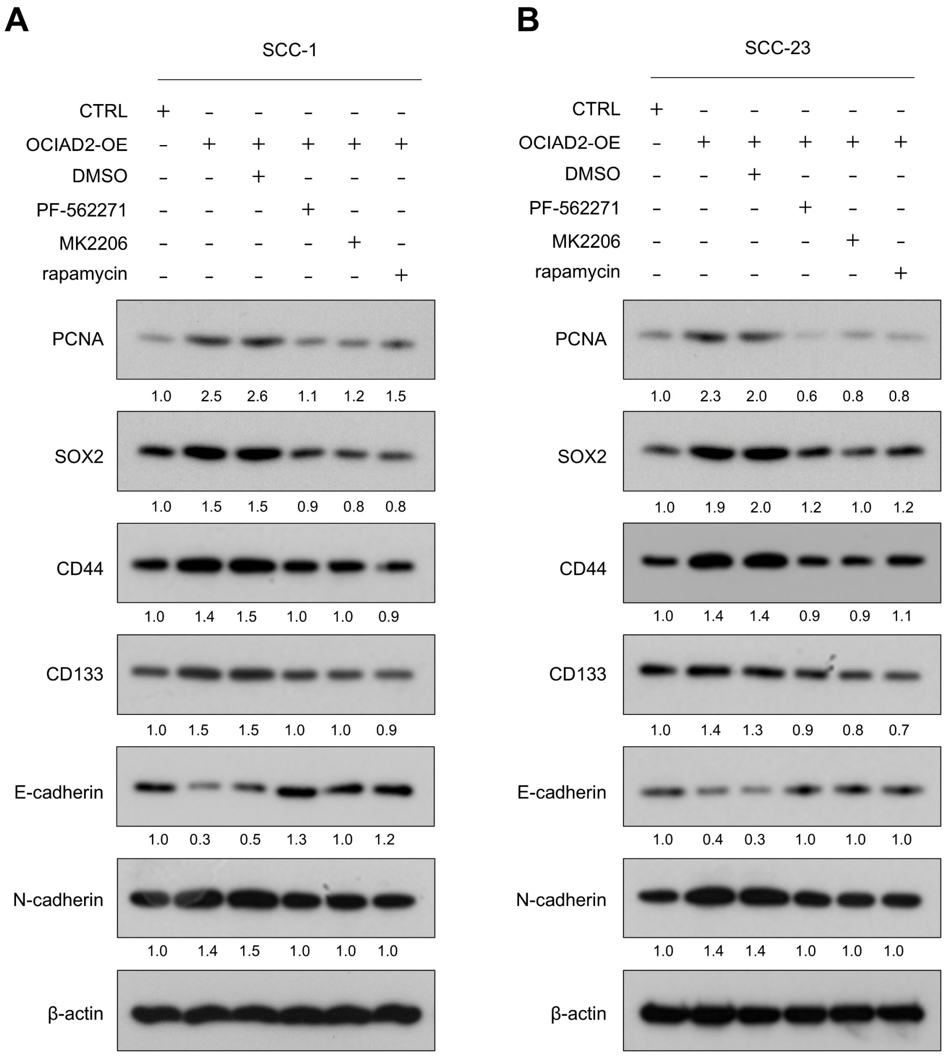

Supplementary Figure 2. **FAK–AKT–mTOR inhibition attenuates OCIAD2-induced expression of stemness- and EMT-related markers.** (A-B) Immunoblot analysis of PCNA, SOX2, CD44, CD133, E-cadherin, and N-cadherin in SCC-1 and SCC-23 cells overexpressing OCIAD2, following treatment with PF-562271, MK2206, or rapamycin (n=3).

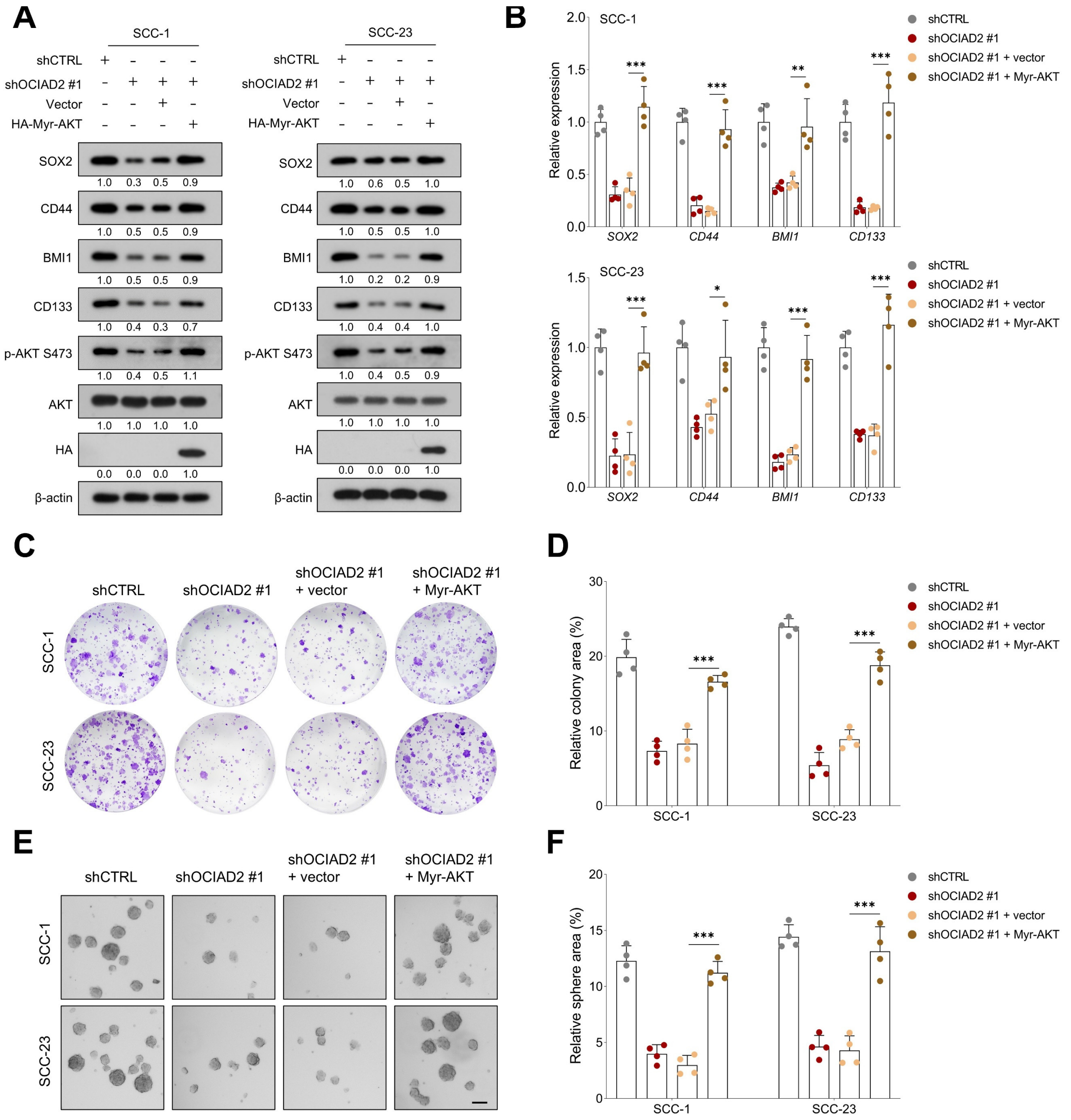

Supplementary Figure 3. Genetic rescue of AKT signaling in OCIAD2-silenced HNSCC cells. (A) Immunoblot analysis of CSC-associated markers SOX2, CD44, BMI1, and CD133, phosphorylated AKT, total AKT, and HA, used as an epitope tag to indicate expression of exogenous HA-Myr-AKT, in SCC-1 and SCC-23 cells expressing control shRNA or OCIAD2-targeting shRNA, with or without transfection of empty vector or constitutively active HA-Myr-AKT (n=3). (B) Quantification of the relative expression of *SOX2*, *CD44*, *BMI1*, and *CD133* under the indicated conditions (n=4). (C–D) Representative images and quantification of clonogenic assays performed in SCC-1 and SCC-23 cells under the indicated genetic conditions (n=4). (E–F) Representative images and quantification of sphere formation assays conducted in SCC-1 and SCC-23 cells under the indicated conditions (n=4). Scale bar, 100 µm. Data are presented as mean ± SD unless otherwise indicated. For comparisons among multiple groups, one-way ANOVA with post hoc multiple-comparisons testing was used. *$P < 0.05$, **$P < 0.01$ and ***$P < 0.001$.

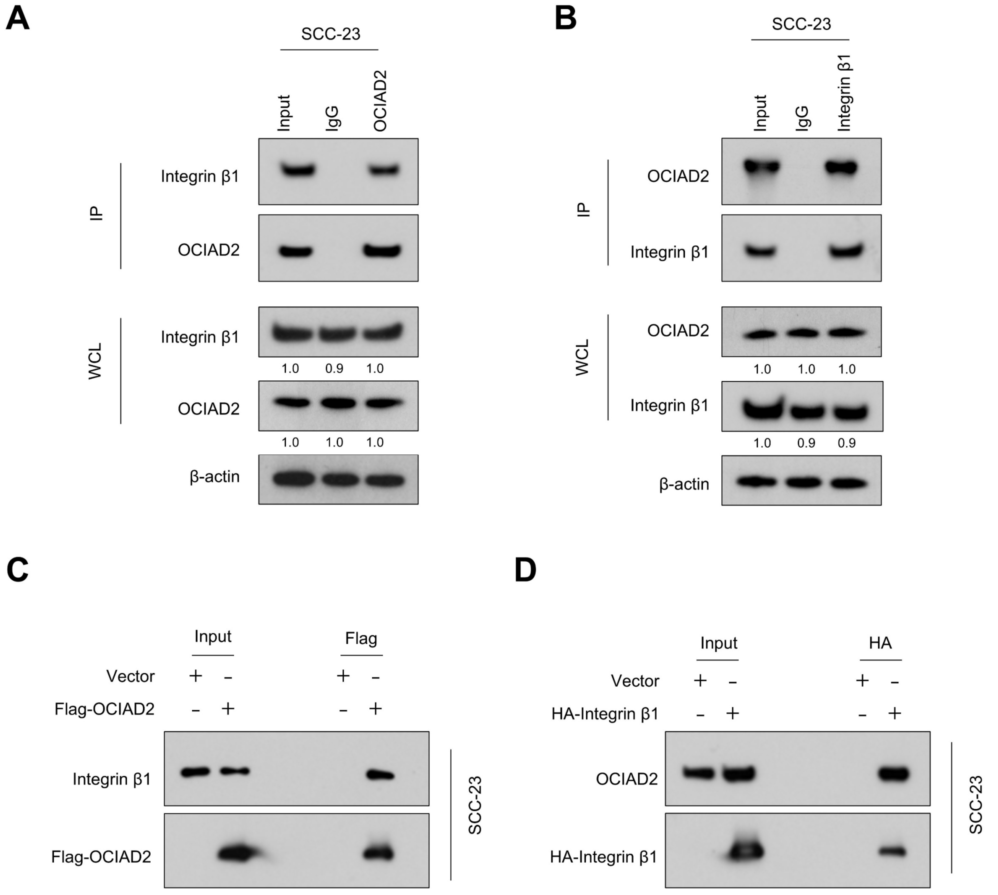

Supplementary Figure 4. **OCIAD2 interacts with integrin β1 in SCC-23 cells.** (A–B) Co-IP assays performed using anti-OCIAD2 or anti–integrin β1 antibodies to assess endogenous protein interactions. (A–B) Co-IP assays in SCC-23 cells using anti-OCIAD2 or anti–integrin β1 antibodies to examine endogenous interactions (n=3). (C–D) Co-IP assays in SCC-23 cells co-expressing Flag–OCIAD2 and HA–integrin β1 using anti-Flag or anti-HA antibodies (n=3).

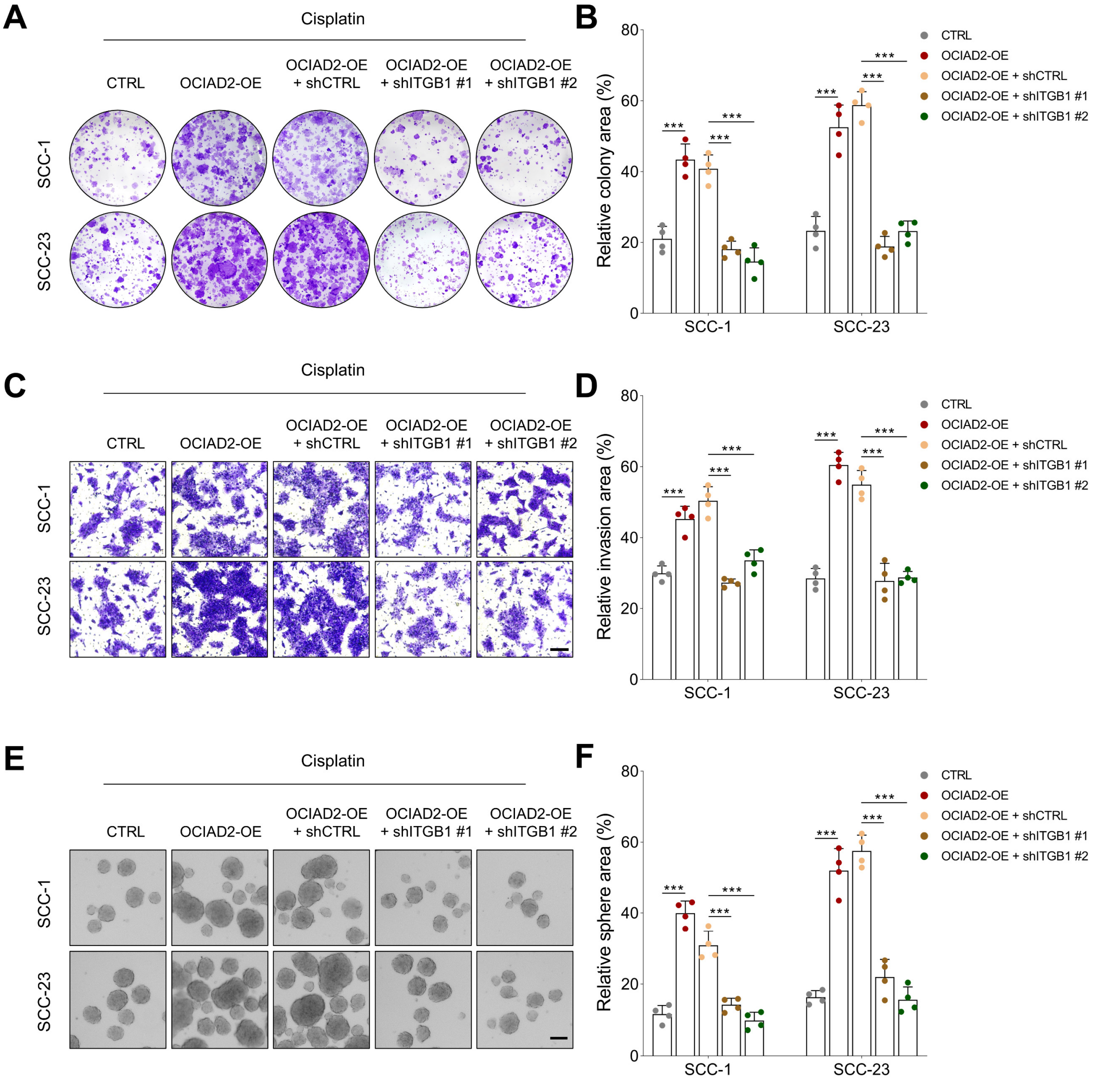

Supplementary Figure 5. Integrin β1 is required for OCIAD2-driven clonogenic growth, invasion, and tumorsphere formation in the presence of cisplatin. (A–B) Clonogenic assays were performed in SCC-1 and SCC-23 cells overexpressing OCIAD2 with or without integrin β1 silencing under cisplatin exposure (n=4). (C–D) Invasive capacity of OCIAD2-overexpressing SCC-1 and SCC-23 cells with or without integrin β1 knockdown assessed by transwell assays under cisplatin treatment (n=4). Scale bar, 100 μm. (E-F) Sphere-forming ability of OCIAD2-overexpressing SCC-1 and SCC-23 cells evaluated under cisplatin treatment with or without integrin β1 depletion (n=4). Scale bar, 100 μm. Data are presented as mean ± SD unless otherwise indicated. For comparisons among multiple groups, one-way ANOVA with post hoc multiple-comparisons testing was used. ****P* < 0.001.

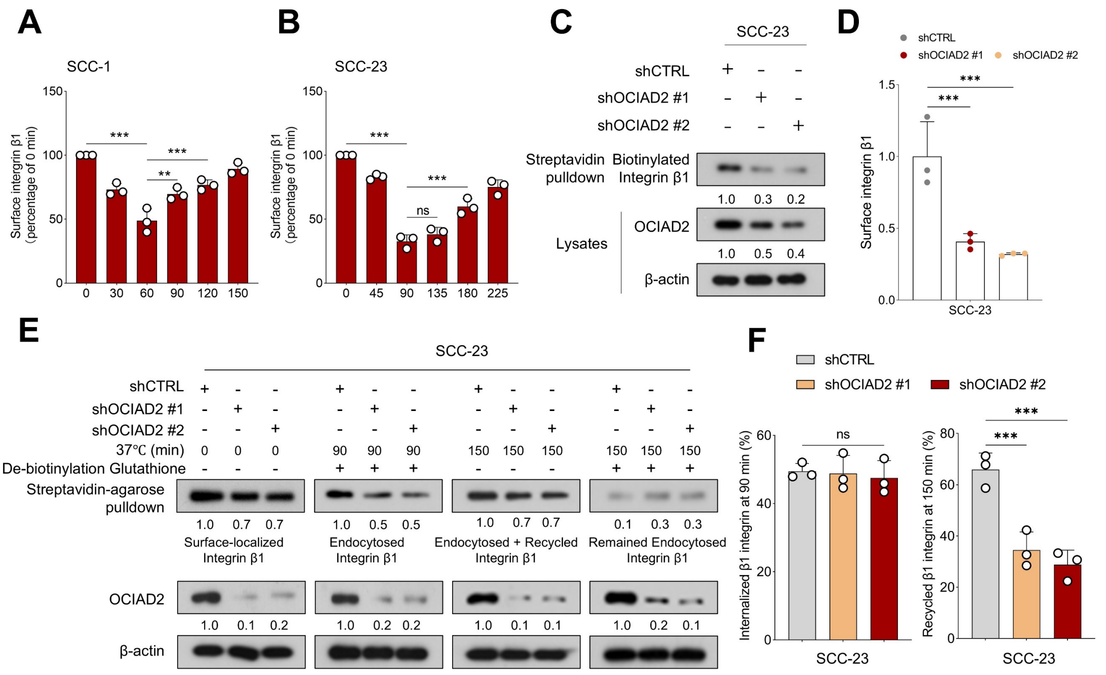

Supplementary Figure 6. **OCIAD2 regulates integrin β1 surface expression and recycling dynamics in HNSCC cells.** (A–B) Flow cytometric analysis of surface integrin β1 levels in HNSCC cells at the indicated time points following nocodazole (NZ) washout (n=3). (C–D) Immunoblot analysis of surface-localized integrin β1 in SCC-23 cells with or without OCIAD2 knockdown, detected by biotinylation and streptavidin pulldown (n=3). (E–F) Biotinylation-based recycling assays evaluating surface, internalized, recycled, and retained integrin β1 in SCC-23 cells following OCIAD2 depletion (n=3). Data are presented as mean ± SD unless otherwise indicated. For comparisons among multiple groups, one-way ANOVA with post hoc multiple-comparisons testing was used. $^{**}P < 0.01$, $^{***}P < 0.001$; ns, not significant.

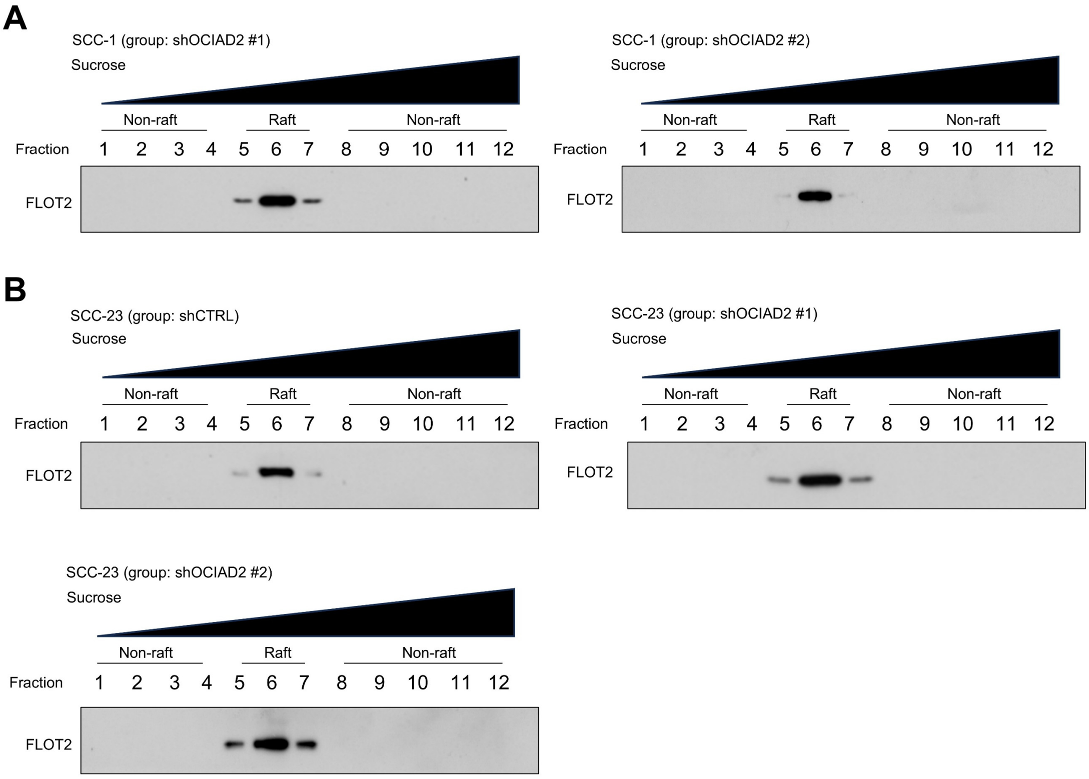

Supplementary Figure 7. Sucrose density gradient fractionation of membrane fractions in HNSCC cells. (A) Immunoblot analysis of FLOT2 across sucrose gradient fractions in SCC-1 cells expressing OCIAD2-targeting shRNAs (n=3). (B) Immunoblot analysis of FLOT2 across sucrose gradient fractions in SCC-23 cells expressing control or OCIAD2-targeting shRNAs (n=3). Cell lysates were subjected to sucrose density gradient ultracentrifugation and fractionated sequentially into 12 fractions. Based on FLOT2 enrichment, fractions 5–7 were designated as lipid raft–associated fractions, whereas fractions 1–4 and 8–12 were classified as non-raft fractions. Raft and non-raft regions are indicated in the schematic above each immunoblot.

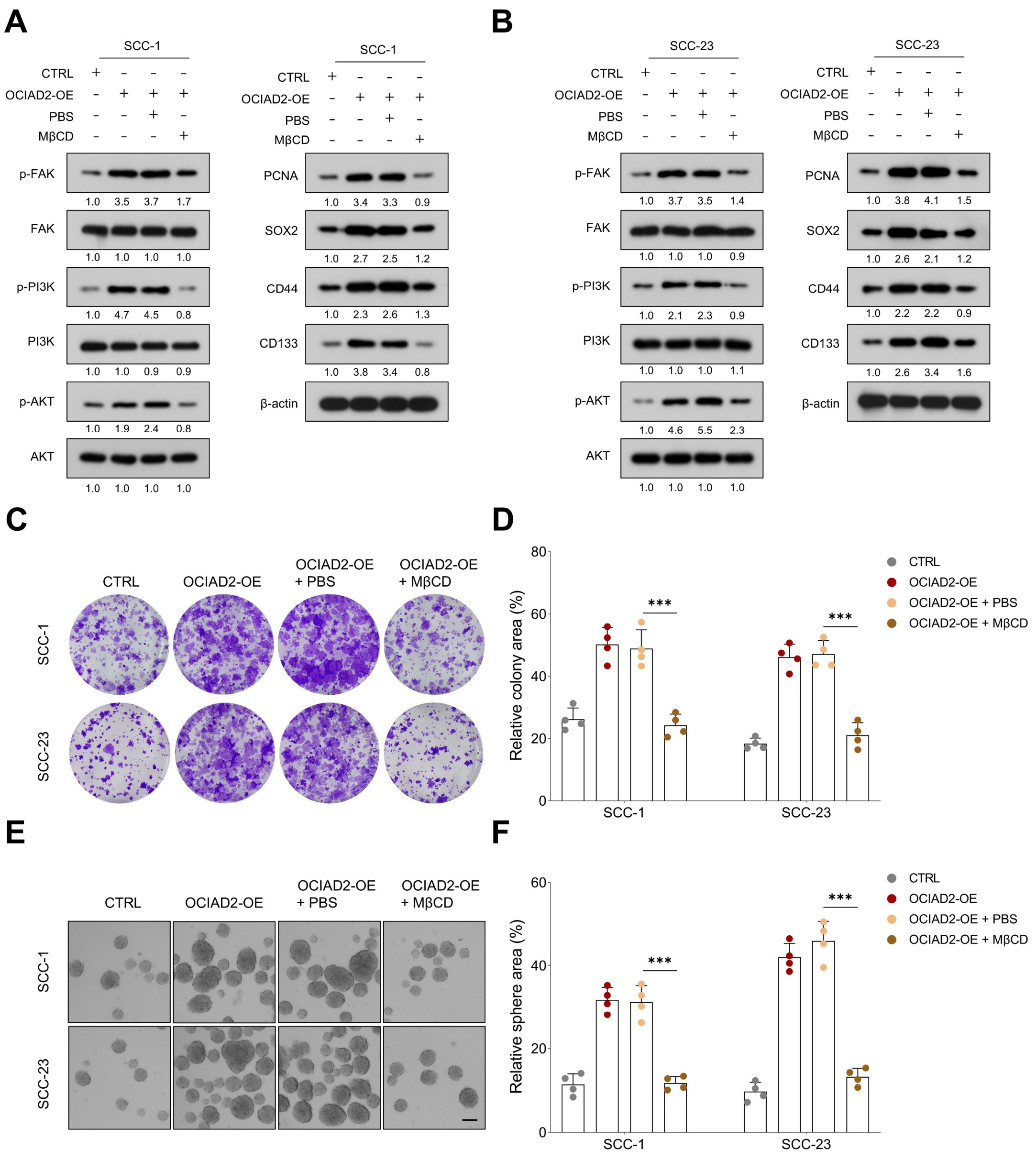

Supplementary Figure 8. Lipid raft disruption attenuates OCIAD2-associated signaling activation and oncogenic phenotypes in HNSCC cells. (A-B) Immunoblot analysis of integrin–FAK–PI3K–AKT signaling components and proliferation- and stemness-associated markers in SCC-1 and SCC-23 cells transduced with control or OCIAD2-overexpressing constructs and treated with PBS or methyl-β-cyclodextrin (MβCD) (n=3). (C-D) Representative images and quantification of clonogenic growth in SCC-1 and SCC-23 cells under the indicated conditions (n=4). (E-F) Representative images and quantification of tumorsphere formation in SCC-1 and SCC-23 cells following OCIAD2 overexpression with or without MβCD treatment (n=4). Scale bar, 100 μm. Data are presented as mean ± SD unless otherwise indicated. For comparisons among multiple groups, one-way ANOVA with post hoc multiple-comparisons testing was used. ***$P < 0.001$.

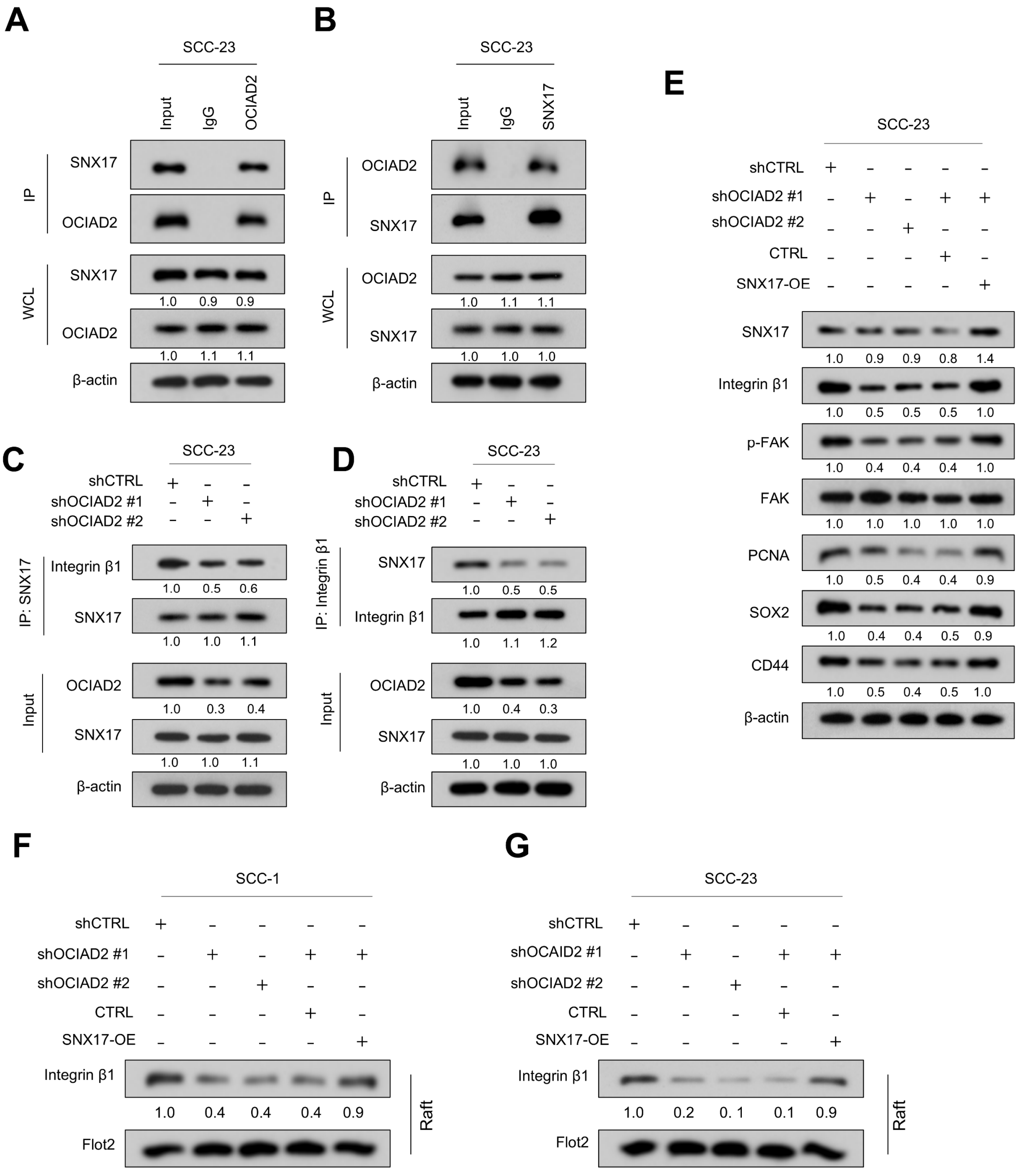

Supplementary Figure 9. SNX17 restores integrin β1 lipid raft localization and downstream signaling following OCIAD2 depletion. (A-B) Co-IP assays in SCC-23 cells using anti-OCIAD2 or anti-SNX17 antibodies to examine the interaction between endogenous OCIAD2 and SNX17 (n=3). (C-D) Co-IP assays assessing the interaction between SNX17 and integrin β1 in SCC-23 cells following OCIAD2 knockdown (n=3). (E) Immunoblot analysis of integrin β1, p-FAK, FAK, PCNA, SOX2, and CD44 in SCC-23 cells transduced with control or OCIAD2-targeting shRNAs, with or without SNX17 overexpression (n=3). (F–G) Western blot analysis of integrin β1 in lipid raft fractions isolated from SCC-1 and SCC-23 cells following OCIAD2 knockdown, in the presence or absence of SNX17 reconstitution (n=3).

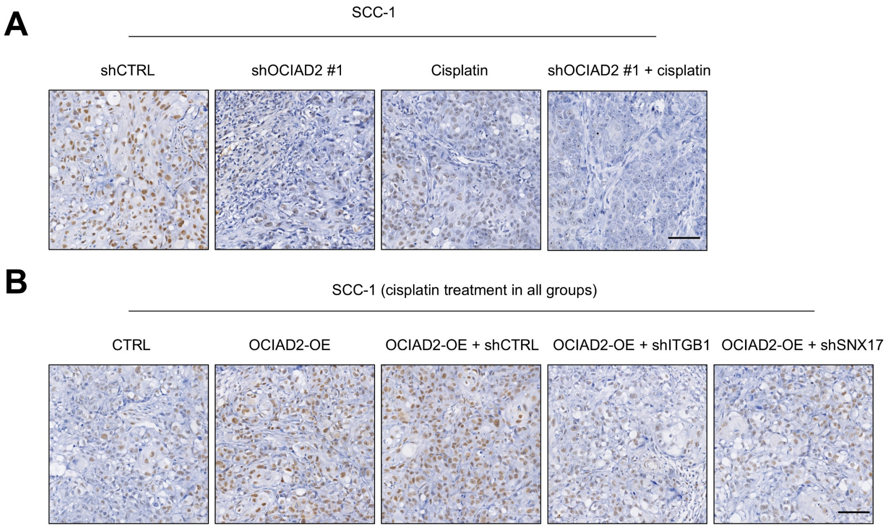

Supplementary Figure 10. Ki-67 staining in tumor xenografts under different treatment conditions. (A) Representative Ki-67 immunohistochemical staining in SCC-1 xenografts derived from cells expressing control or OCIAD2-targeting shRNAs, with or without cisplatin treatment. Scale bar, 100 μm. (B) Representative Ki-67 staining in SCC-1 xenografts derived from cells overexpressing OCIAD2 combined with integrin β1 or SNX17 knockdown under cisplatin treatment. Scale bar, 100 μm.

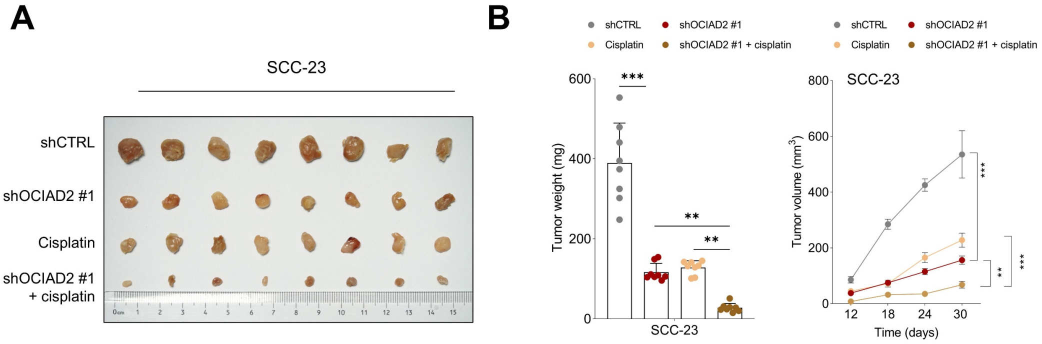

Supplementary Figure 11. OCIAD2 silencing potentiates the antitumor effect of cisplatin in HPV-positive SCC-23 xenografts. (A) Tumors derived from SCC-23 cells expressing control or OCIAD2-targeting shRNA under vehicle or cisplatin treatment (four groups, n=8 mice per group). (B) Tumor weight at endpoint and tumor volume over time in SCC-23 xenografts under the indicated treatment conditions. Data are presented as mean ± SD unless otherwise indicated. For comparisons among multiple groups, one-way ANOVA with post hoc multiple-comparisons testing was used. $^{**}P < 0.01$, $^{***}P < 0.001$.

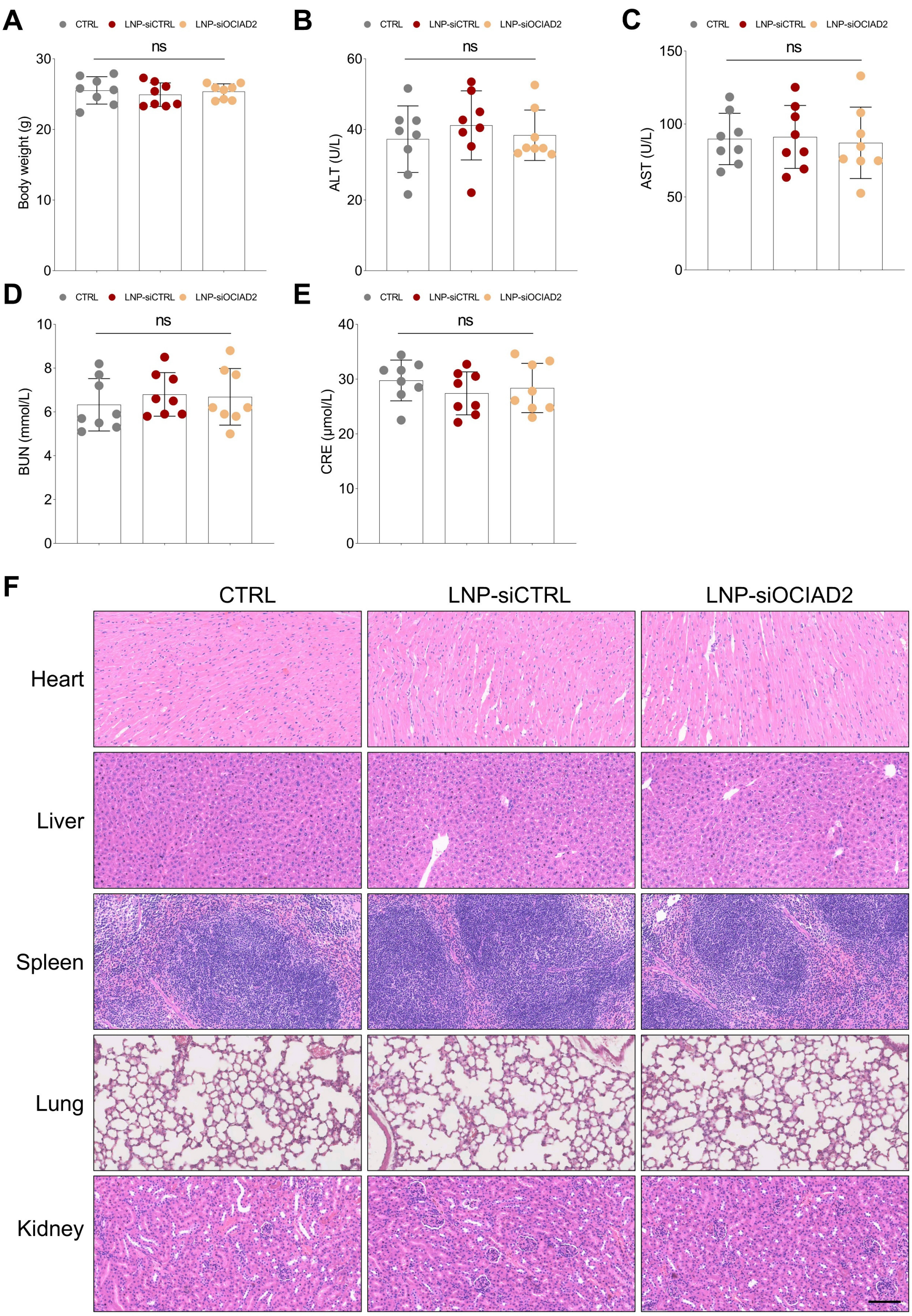

Supplementary Figure 12. In vivo safety evaluation of LNP-mediated siRNA administration. (A) Body weight of mice treated with CTRL, LNP-siCTRL, or LNP-siOCIAD2 (n=8). (B–C) Serum levels of alanine aminotransferase (ALT) and aspartate aminotransferase (AST) (n=8). (D–E) Serum blood urea nitrogen (BUN) and creatinine (CRE) levels (n=8). (F) Representative H&E–stained sections of major organs, including heart, liver, spleen, lung, and kidney, from each treatment group. Scale bar,

100 μm. Data are presented as mean ± SD unless otherwise indicated. For comparisons among multiple groups, one-way ANOVA with post hoc multiple-comparisons testing was used. ns, not significant.

Supplementary Table 1. The clinicopathological characteristics of in-house HNSCC cohort.

Clinicopathological parameters	Number of patients (n, %)
**Age**	
≥60	76 (56.29%)
<60	59 (43.71%)
**Gender**	
Male	106 (78.52%)
Female	29 (21.48%)
**Site**	
Oral cavity	85 (62.96%)
Non-oral cavity	50 (37.04%)
**Smoking status**	
Yes	56 (41.48%)
No	79 (58.52%)
**Differentiation**	
G1-G2	90 (66.67%)
G3	45 (33.33%)
**TNM stage**	
I-II	72 (53.33%)
III-IV	63 (46.67%)

Supplementary Table 2. Sequences of primers and oligos used in this study.

Target / Oligonucleotide	Sequence (5′-3′)
OCIAD2	F: GTCTGCTCGTGGAAACCAAG
	R: CAAGAGACCAGCAAGTGCAAC
ITGB1	F: CCTACTTCTGCACGATGTGATG
	R: CCTTTGCTACGGTTGGTTACATT
β-actin	F: ATCAAGATCATTGCTCCTCCTGAG
	R: CTGCTTGCTGATCCACATCTG
shSNX17	CTATGGTCAAACTCTCAAGTA
shOCIAD2 #1	GAAGAAAGTTTCTGGAAGAGA
shOCIAD2 #2	GAGCAGAGATCTCAAAGATTA
shITGB1 #1	GCCTTGCATTACTGCTGATAT
shITGB1 #2	GCACGATGTGATGATTTAGAA

Supplementary Table 3. The proteins interacting with OCIAD2 identified by mass spectrometry.

UniProt ID	Gene Symbol	Protein Name	Protein_Qscore	Unique_Peptide_Num
O95858	TSPAN15	Tetraspanin-15	486.26	124
Q00325	SLC25A3	Solute carrier family 25 member 3	425.78	86
P35579	MYH9	Myosin-9	371.92	92
P05556	ITGB1	Integrin beta-1	360.77	82
P11586	MTHFD1	C-1-tetrahydrofolate synthase, cytoplasmic	354.69	70
Q9H4M7	PLEKHA4	Pleckstrin homology domain-containing family A member 4	312.48	72
Q00610	CLTC	Clathrin heavy chain 1	298.03	65
P11021	HSPA5	Endoplasmic reticulum chaperone BiP	290.86	62
Q9NX40	OCIAD1	OCIA domain-containing protein 1	275.51	57
P11142	HSPA8	Heat shock cognate 71 kDa protein	246.65	60
